# Comparative Metaproteomic Analysis on Consecutively *Rehmannia glutinosa-*Monocultured Rhizosphere Soil

**DOI:** 10.1371/journal.pone.0020611

**Published:** 2011-05-31

**Authors:** Linkun Wu, Haibin Wang, Zhixing Zhang, Rui Lin, Zhongyi Zhang, Wenxiong Lin

**Affiliations:** 1 School of Life Sciences, Fujian Agriculture and Forestry University, Fuzhou, Fujian, China; 2 Agroecological Institute, Fujian Agriculture and Forestry University, Fuzhou, Fujian, China; 3 College of Oceanography and Environmental Science, Xiamen University, Xiamen, Fujian, China; 4 Institute of Chinese Medicinal Materials, Henan Agriculture University, Zhengzhou, Henan, China; Argonne National Laboratory, United States of America

## Abstract

**Background:**

The consecutive monoculture for most of medicinal plants, such as *Rehmannia glutinosa*, results in a significant reduction in the yield and quality. There is an urgent need to study for the sustainable development of Chinese herbaceous medicine.

**Methodology/Principal Findings:**

Comparative metaproteomics of rhizosphere soil was developed and used to analyze the underlying mechanism of the consecutive monoculture problems of *R. glutinosa*. The 2D-gel patterns of protein spots for the soil samples showed a strong matrix dependency. Among the spots, 103 spots with high resolution and repeatability were randomly selected and successfully identified by MALDI TOF-TOF MS for a rhizosphere soil metaproteomic profile analysis. These proteins originating from plants and microorganisms play important roles in nutrient cycles and energy flow in rhizospheric soil ecosystem. They function in protein, nucleotide and secondary metabolisms, signal transduction and resistance. Comparative metaproteomics analysis revealed 33 differentially expressed protein spots in rhizosphere soil in response to increasing years of monoculture. Among them, plant proteins related to carbon and nitrogen metabolism and stress response, were mostly up-regulated except a down-regulated protein (glutathione S-transferase) involving detoxification. The phenylalanine ammonia-lyase was believed to participate in the phenylpropanoid metabolism as shown with a considerable increase in total phenolic acid content with increasing years of monoculture. Microbial proteins related to protein metabolism and cell wall biosynthesis, were up-regulated except a down-regulated protein (geranylgeranyl pyrophosphate synthase) functioning in diterpenoid synthesis. The results suggest that the consecutive monoculture of *R. glutinosa* changes the soil microbial ecology due to the exudates accumulation, as a result, the nutrient cycles are affected, leading to the retardation of plant growth and development.

**Conclusions/Significance:**

Our results demonstrated the interactions among plant, soil and microflora in the proteomic level are crucial for the productivity and quality of *R. glutinosa* in consecutive monoculture system.

## Introduction

Tuberous root of *Rehmannia glutinosa* Libosch in the family of *Scrophulariaceae* is used as one of the important and highly demanded traditional Chinese medicines. High quality *R. glutinosa* is mainly produced in Jiaozuo, Henan province (35°19′N, 113°51′E) of central China, where the climatic and soil conditions for its cultivation are the most desirable. However, the productivity and quality of the tuberous products substantially decline after consecutive monoculture. This phenomenon is known as soil sickness (replanting disease) [1] and/or consecutive monoculture problems [2]. The consecutively monocultured medicinal plants tend to suffer from severe diseases, which result in reduced biomass, especially decreased tuberous products. To curb the ill-effects, the common practice is to cultivate the medicinal plants only once every eight years on a same lot [3]. Therefore, the farmers have to plant *R. glutinosa* in the less desirable areas outside Jiaozuo, and inevitably have a poor harvest in yield and quality [4].

Previous studies pointed out some possible consequences of the consecutive monoculture, including soil nutrient imbalance, autotoxic substance generation and/or soil borne diseases [4]. For instance, the relative nitrogen (N), phosphorus (P) and potassium (K) contributions to *R. glutinosa*'s biomass production were N>P>K [5]. In addition, the nutritional decline in soil was not the fundamental reason for the ill-effects caused by consecutive monoculture [6]. Rather, the main culprit was believed to be the autotoxicity generated by the plant's root exudates [3], [7]. The GC-MS analysis on the aqueous extracts of *R. glutinosa* rhizosphere soil showed that the allelochemicals, including organic acids, aldehydes and phenolics, were potentially harmful for plant growth [8]. There are reports on the negative effects of phenolic acids on the soil microorganisms, indicating that the allelochemicals (e.g., cinnamic acid, 2,4-di-tert-butylphenol and vanillic acid) can affect the microbial genetic diversity and ecology in soil [1], [2]. More recent studies have revealed that the consecutive monoculture practice influences the structure and populations of *R. glutinosa* rhizoshpere community [9], [10]. However, there is no report hitherto focusing on the relationship among the soil ecosystem, microbial community and consecutive monoculture in a proteomic perspective.

There has been an increasing interest on the biological properties of rhizosphere *in situ* [11]. Various approaches can be utilized to obtain the biological information within rhizosphere. However, the complexity due to the numerous and diverse interactions among the soil's physical, chemical, and biological components seems to have hampered the progress [12]. For example, the microbial biomass carbon [13], microbial respiration [14], lipids [15] and nucleic acids [16] that are frequently used as indicators for the microbial activities, fall short of explaining the functions of soil microbes *in situ*. Metaproteomic analysis is capable of providing information to show the actual functionality with respect to the metabolic reactions and regulatory cascades. The methodology, therefore, may offer a greater potential than the conventional means for functional analysis on the microbial communities.

Metaproteomics is a study of all proteins recovered directly from environmental samples at a given time [17]. It has some superior advantages for soil studies. First of all, the rhizosphere soil metaproteomics provides a direct evidence on the biological processes in soil ecosystem at the proteomic level. Even the nucleic acids-based approaches are greatly restricted due to the weak correlation between mRNA and protein abundances, and the complexity of mRNA post-transcriptional processing and modification [18]. Moreover, the biological process in rhizosphere soil is driven not only by the microbes but also by the plants and fauna in the ecosystem. Extended soil protein identification is essential to an understanding of the soil ecological processes and the environmental factors that affect the functioning of the rhzosphere soil ecosystem [19]. Thus, soil metaproteomics can serve as an indispensable tool for studying rhizosphere biology.

An optimized soil protein extraction protocol was developed and applied in this study. A proteomic comparison between the one- and two-year monoculture soils was performed on the protein extracts obtained. It was speculated that the *R. glutinosa* biomasses, both above- and below-ground, would be reduced when the monoculture was extended from one year to two years, owing to the imbalance among the community members (i.e., plants, microflora and fauna), and the inhibition of nutrient cycling in the rhizosphere soil. In this study, we aimed to: (i) extract proteins directly from the rhizoshpere soil, (ii) determine changes on soil protein abundance under consecutive monoculture, and (iii) understand the interactions between the root system and the rhizospheric microorganisms. The result might provide a theoretical basis and technological support for restoration of soil damage, improvement of soil ecological environment, and establishment of an effective cropping practice to resolve the problems associated with *R. glutinosa* consecutive monoculture.

## Results

### Dried weight of *R. glutinosa* biomasses

Consecutive monoculture significantly inhibited the growth of *R. glutinosa*, as indicated by the reduced above- and below-ground biomasses after the two-year consecutive monoculture (Table 1). The two-year consecutive monoculture significantly lowered the dry weights of the above-ground *R. glutinosa* plant on the 70th, 80th and 90th day after planting, and those of the below-ground tuber on the 60th, 70th, 80th and 90th day after planting. The plant's root to shoot ratios (R/S) on the 50th, 60th, 70th, 80th and 90th day were significantly lowered by the extended monoculture.

**Table 1:** Morphological indices of one- and two-year monocultured *R. glutinosa*.

Days after planting	Years of cultivation	Dry weight (g)		Root to shoot ratio
		aboveground	belowground	
50	One-year^1)^	3.62±0.45a	1.48±0.39a	0.38
	Two-year^2)^	5.32±0.38a	1.47±0.27a	0.31
60	One-year	5.53±0.22a	6.62±0.26a	1.16
	Two-year	6.81±0.35a	3.42±0.53b	0.41
70	One-year	8.71±0.44a	9.91±0.47a	1.14
	Two-year	8.14±0.29b	2.72±0.36b	0.35
80	One-year	12.19±0.43a	18.94±0.55a	1.55
	Two-year	8.53±0.51b	4.61±0.28b	0.5
90	One-year	17.57±0.31a	25.08±0.35a	1.43
	Two-year	9.85±0.29b	7.51±0.53b	0.8

Note: Data are means±SE (*n* = 10), and different letters show significant differences at the 5% level according to its p-value between *R. glutinosa* samples harvested on different length of time after planting for one-year monoculture^1)^ and two-year consecutive monoculture^2)^ by Tucky's test (*P*≤0.05).

### Root activity and N, P and K contents of *R. glutinosa*

The consecutive monoculture significantly retarded the *R. glutinosa*'s root activity, as indicated by reductions of the plant's bleeding intensity (BI) and K content in the sap (Table 2). From 95 to 135 days after planting, the plant's BI was consistently higher in the one-year than that in the two-year consecutive monocultured plants. The changes in the sap's K content had a similar trend. N and K contents in *R. glutinosa* significantly declined under the two-year consecutive monoculture (Table 3). On the other hand, there was no significant difference in the P content between the one- and two-year monocultured samples.

**Table 2:** Effects of consecutive monoculture on *R. glutinosa* root activity.

Days after planting	Years of cultivation	Bleeding intensity (mg/h)	Potassium (mg/ml)
95	One-year^1)^	31.6a	0.07a
	Two-year^2)^	2.25b	0.01b
110	One-year	14.7a	0.33a
	Two-year	6.01b	0.26b
135	One-year	9.16a	0.27a
	Two-year	6.32b	0.20b

Note: Different letters show significant differences at the 5% level according to its p-value between *R. glutinosa* samples harvested on different length of time after planting for one-year monoculture^1)^ and two-year consecutive monoculture^2)^ by Tucky's test (n = 10, *P*≤0.05).

**Table 3:** Effects of consecutive monoculture on N, P and K contents in *R. glutinosa.*

Days after planting	Years of cultivation	N (mg/g)	P (mg/g)	K (mg/g)
70	One-year^1)^	14.3a	1.86a	3.89a
	Two-year^2)^	15.4a	2.00a	3.17b
95	One-year	11.3a	1.51a	3.19a
	Two-year	6.80b	1.66a	2.00b
110	One-year	12.7a	2.16a	2.87a
	Two-year	10.8b	1.77a	2.61b

Note: Different letters show significant differences at the 5% level according to its p-value between *R. glutinosa* samples harvested on different length of time after planting for one-year monoculture^1)^ and two-year consecutive monoculture^2)^ by Tucky's test (n = 10, *P*≤0.05).

### Total phenolic acids in rhizospheric soils

Consecutive monoculture had a significant effect on the content of total phenolic acids in soil (Table 4). The total phenolic acid content was significantly higher in the two-year than in the control or the one-year monocultured soil. In addition, the inhibition effect of the phenolic acid extract (PAE) from the soil samples on lettuce's root length showed a significantly greater retardation on the root growth in the two-year monoculture, as compared to the control and that in the one-year monoculture.

**Table 4:** Effects of consecutive monoculture on total phenolic acids in soil.

	Unplanted soil	One-year monoculture soil	Two-year monoculture soil
Total phenolic acids in soil (μmol/g)	7.49±0.13c	8.64±0.15b	9.45±0.42a
Length of lettuce root (cm)	2.64±0.23a	2.49±0.27a	2.07±0.26b
Lettuce root growth inhibition rate	0%	5.7%	21.5%

Note: Data are means±SE, and different letters show significant differences at the 5% level according to its p-value between total phenolic acid contents by Tucky's test (P≤0.05, n = 3) and lengths of lettuce root by Tucky's test (P≤0.05, n = 50).

### Profile analysis of metaproteome in rhizospheric soils

**Figure 1:**
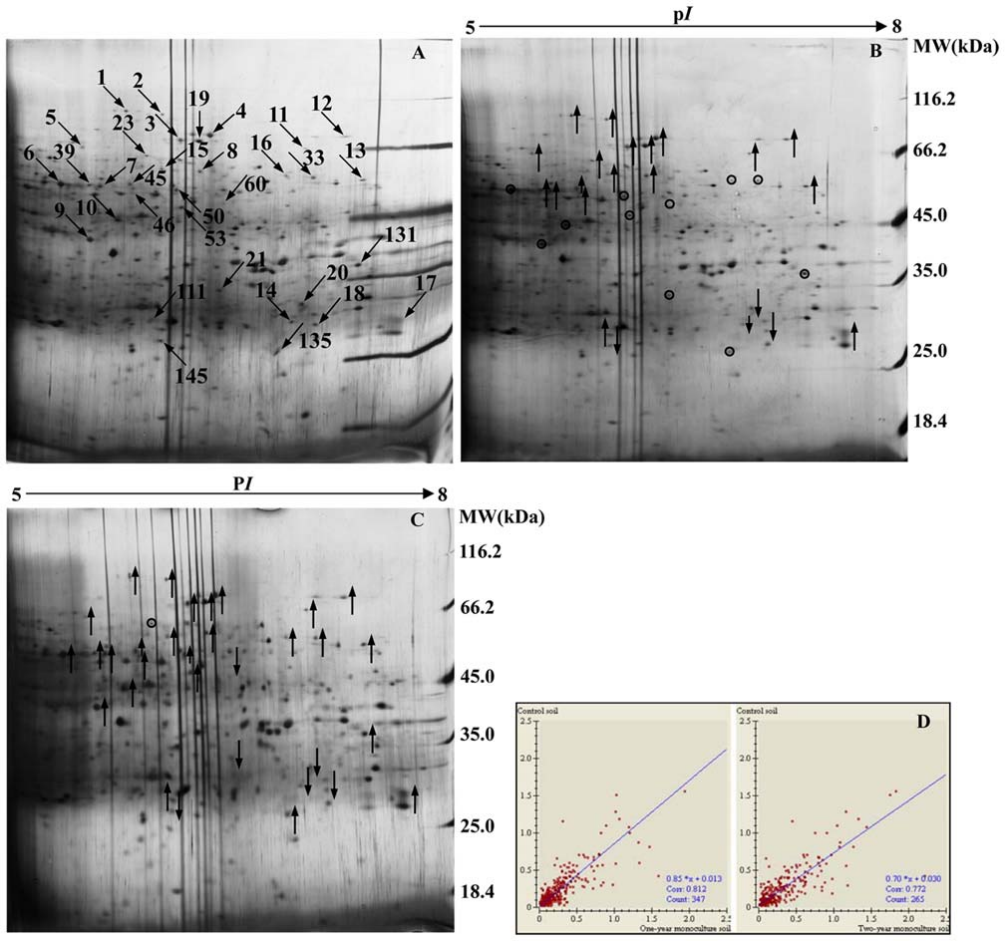
Silver stained 2-D gel of proteins extracted from rhizosphere soil. **A:** Proteins extracted from the control soil. **B:** Proteins extracted from the one-year *R. glutinosa*-monocultured soil; **C:** Proteins extracted from the two-year *R. glutinosa*-monocultured soil. **D:** Repeatability analysis of 2-DE maps of soil proteins extracted from three different soil samples. Arrows in A point at proteins with differential expressions. Upward arrows in B and C indicate the positions of up-regulated proteins and downward arrows show the positions of down-regulated proteins, while white circles in B and C represent the same expression level compared to the control. Scatter plots in D give an idea of the relationship between the spot values (%Vol) from two gels (CK vs NP, CK vs CM) by searching for the linear dependence between the spot values of one gel (variable X, namely NP or CM) and the corresponding values in a reference gel (variable Y, namely CK).

Further analysis was done to investigate the changes of the proteins from rhizosphere soil samples in response to the consecutive monoculture. A high resolution 2-DE gel protein separation was applied in the p*I* range between 5 and 8. After silver staining, protein spots were isolated and analyzed using the ImageMaster™ 2D Platinum software (version 5.0, GE Healthcare, Uppsala, Sweden). Highly reproducible 2-DE maps were obtained in the three different soil samples with significant correlations of scatter plots (Figure 1). The correlation index between the control soils and the one-year monoculture soils, and the correlation index between the control soils and the two-year monoculture soils were 0.772 and 0.812, respectively. All 2-DE images had a similar spot distribution pattern, implying that they shared similar bio-information.

**Figure 2:**
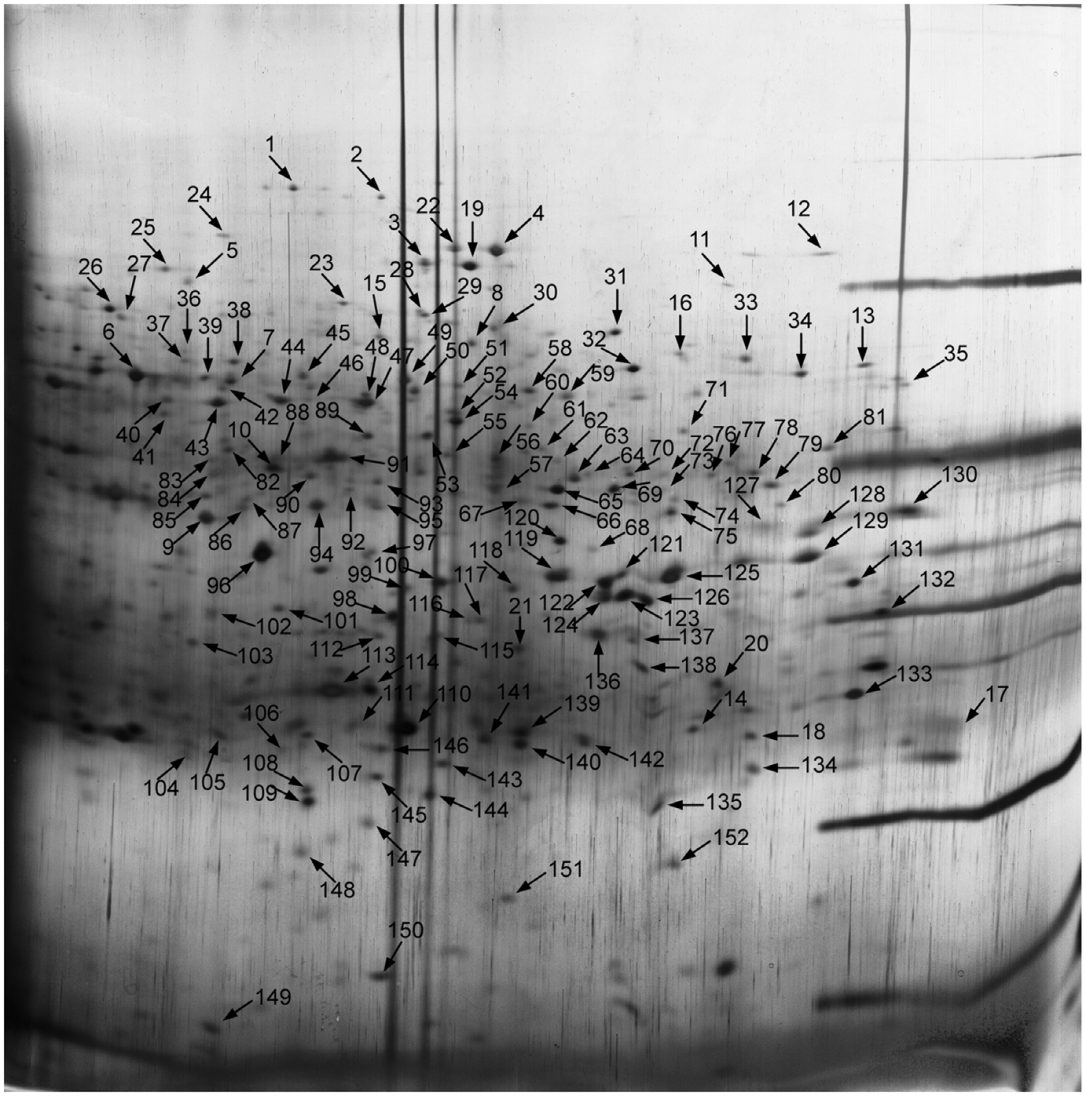
Representative 2-DE gel of proteins extracted from control. Spot numbers correspond to numbers used in Table S1, S2 and S3.

**Figure 3:**
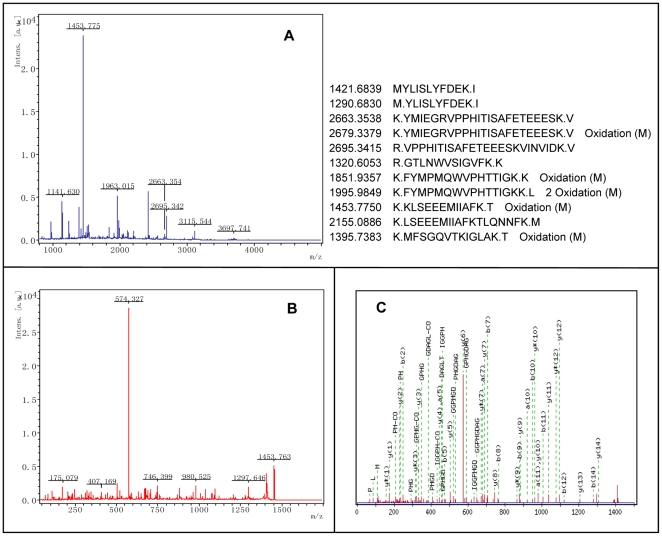
Representative MS spectra of proteins identified by MALDI TOF-TOF MS. Protein Spot 10 was excised from gels, and spectrum of peptides derived after tryptic digestion. (A) MS spectrum of Ion 1453.775 was analyzed by MS/MS. (B) TOF/TOF spectrum of Ion 1453.775. (C) Tandem mass spectrum that confirmed the responding amino acid sequence, FVIGGPHGDAGLTGR, by analyzing b- and y-ions derived from the peptide ion.

To obtain a metaproteomic profile for the *R. glutinosa* rhizosphere soil, 152 protein spots with high resolution and repeatability were randomly selected and excised from the prepared gels, digested in-gel with trypsin, and 103 protein spots were successfully analyzed by LIFT-MALDI TOF-TOF MS (Figure 2, 3). This meant that 49 protein spots failed to be identified by MS maybe due to the excision process not producing usable data or the incomplete environmental metaproteome databases. Database searching was conducted with the BioTools 3.1 software and MASCOT 2.2.03 search engine, firstly against all entries on NCBInr and followed by the ‘Bacteria’ and ‘Fungi’ database. Thirty-four proteins sharing equal searching by MS/MS and MS against all entries are listed in Table S1. Forty-one proteins matched at least 2 MS/MS peptides are listed in Table S2. Twenty-eight proteins matched at least 3 peptide mass fingerprintings (PMFs) are listed in Table S3.

**Figure 4:**
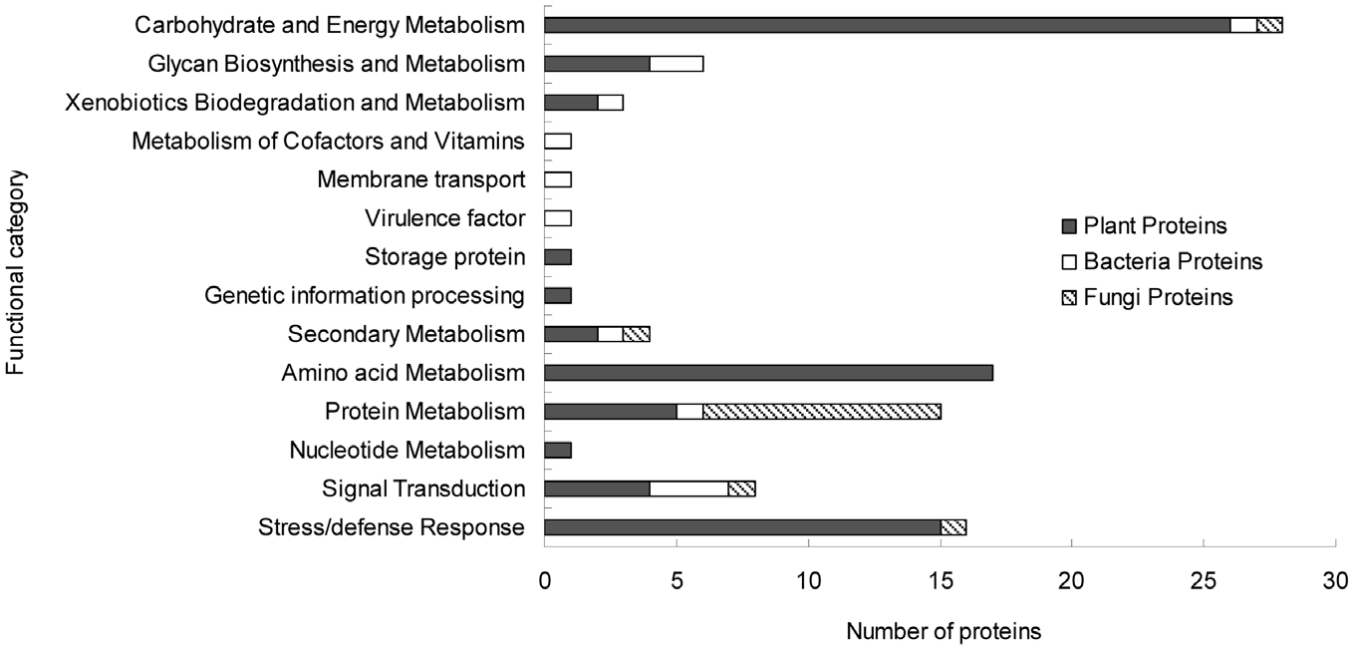
Functional classification of identified proteins. Identified proteins were classified according to their functions using KEGG database (Kyoto Encyclopedia of Genes and Genomes, http://www.genome.jp/kegg/).

The identified proteins were classified by their functions using the KEGG database (Kyoto Encyclopedia of Genes and Genomes at http://www.genome.jp/kegg/). According to the putative physiological functions, they were categorized into 14 groups, as shown in Figure 4, by their association with (i) carbohydrate and energy metabolism, (ii) glycan biosynthesis and metabolism, (iii) xenobiotics biodegradation and metabolism, (iv) cofactors and vitamins metabolism, (v) secondary metabolism, (vi) amino acid metabolism, (vii) protein metabolism, (viii) nucleotide metabolism, (ix) signal transduction, (x) stress/defense response, (xi) genetic information processing, (xii) storage protein, (xiii) virulence factor and (xiv) membrane transport. Among them, 75.73% were derived from plants, 11.65% from bacteria and 12.62% from fungi (Table S1, S2 and S3). It demonstrated that the chemical-biological process in the rhizosphere ecosystem is driven by both the plants and the microbes, and even by the fauna. The largest functional group was the proteins involved in carbohydrate and energy metabolism (27.18%), followed by those associated with the amino acid metabolism (16.50%). They were associated with the soil nutrient cycles, including carbon (C) and N cycling. Sixteen proteins related to the stress/defense response (including the superoxide dismutase and catalase), 4 involved in the secondary metabolism (including the phenylalanine ammonia-lyase, geranylgeranyl pyrophosphate synthase and Pentalenene synthase), and 3 related to the xenobiotics metabolism (including the glutathione S-transferase and tellurite resistance protein) were also identified. Eight protein spots (including the chemotaxis signal transduction protein, methyl-accepting chemotaxis protein, GTP-binding protein, G-protein signaling regulator and TGF-beta receptor-interacting protein 1) from both the microbes and the plants relating to the signal transduction were detected.

Based on the metaproteomic data, a tentative metabolic model for the rhizosphere soil proteins was proposed as shown in Figure S1. It indicated the complex interrelationship among the diverse metabolism pathways. The identified soil proteins function in energy production, protein, nucleotide, amino acid and secondary metabolisms, membrane transport, signal transduction and resistance, etc. Most of proteins involved in the carbohydrate and amino acid metabolism originated from plants, which might provide the energy necessary and precursor materials for the organic acid efflux process, secondary metabolism and defence responses under biotic and abiotic stresses. However, some microbe proteins related to the membrane transport including the ABC transporter ATP-binding subunit, termed quorum-sensing regulated transporter, and signal transduction including the chemotaxis signal transduction protein and methyl-accepting chemotaxis protein were identified in the rhizospheric soil, which might play an important role in the root colonization of microbes. Therefore, it was clear that soil proteins from both the plants and the microbes played roles in the rhizosphere biological process through the primary metabolism, secondary metabolism, signal transduction, etc. They influenced the nutrient cycling in the rhizosphere ecosystem and mediated the interactions between the plants and the soil microbes (Table S1, S2 and S3).

### Differentially expressed proteins and their functions in rhizospheric soils

**Figure 5:**
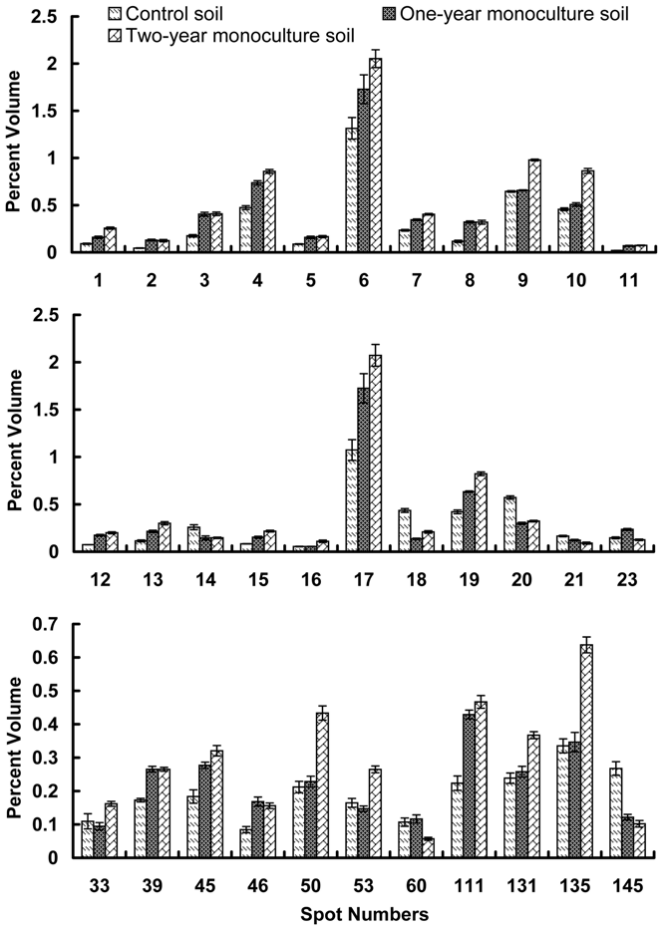
Expression levels of 33 identified proteins as compared to control. Changes in protein expression under consecutive monoculture were calculated by Image Master software 5.0.

Since the soil proteins have a significant effect on the soil biological process, a comparative analysis was carried out to analyze the changes of metaproteomic characterization in the consecutively *R. glutinosa*-monocultured rhizosphere soil. A quantitative analysis revealed that a total of 33 protein spots with high repeatability were differentially expressed, i.e., their intensities varied, at least on one gel in comparison to the control, by more than 1.5-fold. Among the differentially expressed proteins, 4 spots (spots 14, 18, 20 and 145, constituting 12.12%) were down-regulated with the increasing years of monoculture. Nine spots (spots 6, 9, 10, 16, 33, 50, 53, 131 and 135, constituting 27.27%) were up-regulated only in the two-year monoculture soil, but none in the one-year monoculture soil. One spot (spot 23, constituting 3.03%) was up-regulated only in the one-year monoculture soil, but none in the two-year monoculture soil. Two spots (spots 21 and 60, constituting 6.06%) were down-regulated only in the two-year monoculture soil, but none in the one-year monoculture soil. The remainders (spots 1, 2, 3, 4, 5, 7, 8, 11, 12, 13, 15, 17, 19, 39, 45, 46 and 111, constituting 51.52%) were all up-regulated with the increasing years of monoculture (Figure 5).

As shown in Table S4, 33 protein spots with differential expressions were successfully identified by MS. Among them, spot 1 and 2, spot 3 and 19, and spot 14 and 18 were found to be two identical proteins in function. These 26 proteins from the plants (constituting 78.79%) were sorted into 8 categories according to their functions using the KEGG database: (i) carbohydrate metabolism and energy (spots 1, 2, 5, 6, 8, 9, 11 16, 23 and 60, constituting 38.46%), (ii) glycan metabolism (spot 12, constituting 3.85%), (iii) amino acid metabolism (spots 4, 7, 10, 13, 15 and 46, constituting 23.08%), (iv) protein metabolism (spot 39, constituting 3.85%), (v) stress/defense response (spots 17, 33 and 131, constituting 11.54%), (vi) xenobiotics biodegradation and metabolism (spots 14 and 18, constituting 7.69%), (vii) secondary metabolism (spots 3 and 19, constituting 7.69%), and (viii) signal transduction (spot 21, constituting 3.85%). The largest functional category was the proteins involved in carbohydrate metabolism and energy (including the glyceraldehyde-3-phosphate dehydrogenase and aconitate hydratase), followed by those associated with the amino acid metabolism (including the serine hydroxymethyltransferase and S-adenosylmethionine synthetase). In addition, two protein spots representing the same protein (phenylalanine ammonia-lyase) involved in the secondary metabolism and three in the stress/defense response (including the superoxide dismutase and catalase) were also identified. These 7 proteins from the microbes (constituting 21.21%) were categorized into 6 groups: (i) glycan metabolism (spot 53, constituting 14.29%), (ii) cofactors and vitamins metabolism (spot 111, constituting 14.29%), (iii) protein metabolism (spots 45 and 135, constituting 28.57%), (iv) secondary metabolism (spot 20, constituting 14.29%) (v) signal transduction (spot 145, constituting 14.29%) and (vi) virulence factor (spot 50, constituting 14.29%). In sum, consecutive monoculture induced the changes of the expression of soil proteins from both the plants and the microbes. These differentially expressed soil proteins participated in the primary metabolism, secondary metabolism and stress/defense response, etc.

## Discussion

To date, this is the first report using comparative soil metaproteomics to study the effects involving consecutive monoculture. The result might help to unravel the intricate interactions among plant root system, root exudates and rhizospheric microflora. In the present study the identified proteins of plants, bacteria and fungi were involved in several metabolic pathways such as the energy production, protein biosynthesis and turnover, xenobiotics biodegradation, defence machinery and secondary metabolism. Most of these pathways were associated with the soil nutrient cycles, including C and N cycling [20]. Recent advances have shown that low-molecular-weight (LMW) organic compounds in the rhizosphere have a specific role in plant-microbe-soil interactions [11]. Signal molecules exchanged between plants and microorganisms have been identified that favor beneficial plant colonization [21]. Plant roots might develope defence strategies by secreting compounds into the rhizosphere that interfere with bacterial quorum sensing responses [22]. However, motile bacteria could respond to environmental cues or a specific plant to move to more favorable locations [23]. In the present study, several proteins from plants and microbes relating to the signal transduction were detected in the rhizosphere soil. These proteins might play a vital role in the cross-talking process and induce metabolic changes inside the organisms. Two soil proteins (including the chemotaxis signal transduction protein and methyl-accepting chemotaxis protein) related to the signal transduction were detected in this study, which controlled the chemotaxis behaviors of bacteria. Comparative metaproteomic analysis of the consecutively monocultured *R. glutinosa* rhizosphere soil revealed that consecutive monoculture had a profound effect on the metaproteomic characterization in the rhizosphere soil and altered the expression level of soil proteins from both plants and microbes. These differentially expressed proteins were related to various metabolic pathways such as carbohydrate/energy metabolism, amino acid metabolism, secondary metabolism, stress/defense response etc.

### Carbohydrate/energy metabolism and root exudates

Our proteomic analysis showed that 7 proteins (spots 1, 2, 5, 6, 8, 9 and 16) derived from the plants linked to the glycolysis (EMP) / tricarboxylic acid (TCA) cycle, and were highly expressed in the two-year monoculture soil. Release of low-molecular-weight (LMW) organic compounds from the plant roots could be affected by a wide range of stress conditions, such as toxic microbial metabolites [24], [25], low pH of soil [26], [27], extreme temperatures [28], drought [29] and nutrient deficiency [30]. In a consecutive monoculture, *R. glutinosa* releases a large number of LMW root exudates including sugars, carboxylates, amino acids and phenolics [7], [31]–[33]. Some researchers suggested that the increased efflux was not the cause of the slowed growth; rather, slow growth led to the increased efflux [34]; and hence, the raised respiratory cost [35]. However, the passive efflux needs to be compensated by an increased active influx, and therefore, a heightened respiratory or energy cost for the plant. Furthermore, rhizosphere C input from plant roots via rhizodeposition was the driving force for the well-documented ‘rhizosphere effect’, which stimulated microbial growth and activity in close proximity to plant roots [36]. Increased microbial activity in the rhizosphere could promote competition between microbes and plants for limiting mineral nutrients such as N, P, Fe and Mn [37]. Some reports, on the other hand, suggested that the root exudation was not so much a passive event, but a means of manipulating the C content in the rhizosphere that changes soil microbial population [38]. Increased rhizodeposition has been reported as a response to a wide range of nutrient limitations, such as deficiencies in P, K, calcium or zinc [30], [39]. Carboxylate exudates, such as malate, fumarate, oxalate, malonate, citrate and aconitate, are substances associated with P mobilisation [35], [40], which are mostly the intermediates of the TCA cycle.

### Amino acid metabolism and root exudates

Six proteins (spots 4, 7, 10, 13, 15 and 46) originated from the plants linked to the amino acid metabolism were up-regulated, and two (spots 14 and 18) down-regulated. The up-regulated enzymes are catalysts in plant's N metabolism. They might lead to the release of amino acids in the plant causing partially the increased amino acid concentration in the roots [41]. Amino acid metabolism is also essential for protein metabolism. Most of the differentially expressed proteins from the plants were up-regulated as observed in the present study (Figure 5 and Table S4). Carbon and N metabolisms are linked by the shared intermediates and products [38], [42]. Plants can release either amino acids, or organic acids converted from amino acids. The protein, spot 7, was identified as methylmalonate-semialdehyde dehydrogenase, which involves in the propanoate metabolism. This enzyme catalyzes the conversion of methylmalonate semialdehyde (catabolite of valine) into propanoyl-CoA, which could be further converted into one of the LMW organic acids, propanoate, by propanoyl-CoA transferase.

The down-regulated proteins (spots 14 and 18) was identified as glutathione S-transferase (GST), which was linked to detoxification function in the plants. It catalyzes a variety of reactions, and accepts endogenous and xenobiotic toxic substrates [43]. A study analyzed the effects of plant polyphenols on the uncharacterized rat liver GSTs, and reported that several novel naturally occurring flavonoids and other polyphenols exerted varying degrees of concentration-dependent inhibition on GST [44]. Therefore, GST was down-regulated possibly due to accumulation of phenols in the consecutive monocultured rhizosphere. As a consequence, the plant could not effectively detoxify toxins, further worsening the autotoxic effect.

### Secondary metabolism and allelopathy

Two proteins, spot 3 and spot 19, linked to the secondary metabolism were found to be highly expressed with the extended monoculture. The two separate spots (spots 3 and 19) originated from the plants were identified to be the same protein, phenylalanine ammonia-lyase. The enzyme catalyzes the deamination of phenylalanine into cinnamate and ammonia, which is the first step in the formation of phenylpropanoids. The products of phenylpropanoid metabolism, such as coumaric acid, ferulic acid, 4-hydroxy-benzoate, vanillin, etc., were allelochemicals that are detrimental to plant development [7], [33], [45], [46]. In the present study, total phenolic acid content was found to be significantly higher in the two-year than the one-year monoculture soil (Table 4).

### Stress/defense response proteins

Our proteomic analysis showed that two proteins (spots 17 and 33) originated from the plants relating to the stress/defense response were highly expressed in the two-year monoculture soil. They were identified as superoxide dismutase and catalase, important agents for antioxidant defense in nearly all cells [47], [48]. One up-regulated protein (spot 131) was identified as ricin B-related lectin (previously called type 2 ribosome- inactivating proteins, RIPs), which plays an important role in plant interactions with pest insects [49]. One protein (spot 39) from the plants relating to the protein folding, namely mitochondrial chaperonin-60 was also highly expressed with the increasing years of monoculture. The chaperonins, a ubiquitous family of sequence-related molecular chaperones, are essential for protein folding under both normal and stressful conditions [50], [51]. However, 6-phosphogluconate dehydrogenase (spot 60), a protein related to the pentose phosphate pathway was down-regulated in the two-year monoculture soil. This pathway is a process that generates NADPH and pentoses. One of the functions of NADPH in the cell is to prevent oxidative stress [52]. As the plants are under consecutive monoculture conditions, they are faced with stresses such as autotoxic allelochemicals accumulation soil borne diseases. Our results suggested that, the plant might prevent oxidative stress through superoxide dismutase and catalase rather than NADPH generated in the pentose phosphate pathway.

### Soil proteins originated from microbes

Six proteins from the bacteria (spots 20, 45, 50, 53, 111 and 145) and 1 from the fungi (spot 135) were differentially expressed with the extended monoculture. Among them, 4 proteins (spots 45, 53, 111 and 135) were highly expressed in the two-year monoculture soil. They were identified as threonyl-tRNA synthetase, cellulose synthase regulator protein, lipoyl synthase and mitochondrial ribosomal protein L8, which were related to the protein metabolism [53], cell wall biosynthesis [54], cofactors and vitamins metabolism [55], and mitochondrial protein metabolism [56], respectively. These metabolism pathways play important roles in the life process such as the growth, reproduction and heredity of the bacteria. One up-regulated protein (spot 50) was identified as filamentous hemagglutinin (FHA), which has been reported as a bacterial virulence factor required for plant tissue colonization being mainly involved in surface attachment and biofilm formation [57]. However, one down-regulated protein (spot 145) was identified as methyl-accepting chemotaxis protein, which is a sensory protein important in chemotaxis of numerous beneficial bacteria, i.e. *Sinorhizobium meliloti* [58]. Using a process termed chemotactic response, motile bacteria are capable of detecting numerous attractants and repellents and responding appropriately by moving towards increasing concentrations of nutrients and away from increasing concentrations of toxic compounds [59]. In the present study, we also found that one protein (spot 20) was down-expressed with the increasing years of monoculture, identified as geranylgeranyl pyrophosphate synthase originated from *Mycobacterium intracellulare*. This enzyme involves in the terpenoid pathway. It catalyzes the condensation of famesyl-diphosphate and isopentenyl-diphosphate to form geranylgeranyl diphosphate. Geranylgeranyl diphosphate is the synthetic precursor of diterpenoid backbone, including gibberellins (GAs). GAs are a group of diterpenoid acids that function as plant growth regulators that modulate various developmental processes, including stem elongation, germination, dormancy, flowering, sex expression, enzyme induction, and leaf and fruit senescence in a plant. It is likely that consecutive monoculture could alter the root exudates composition, which leads to population decline for the beneficial plant growth-promoting rhizospheric microorganisms (PGPRs). PGPRs are a heterogeneous group of microbes that stimulates plant growth through various mechanisms, such as plant hormone production [60], N_2_ fixation [61], K solubilisation [62] and pathogen suppression [63], and stimulation of other beneficial microorganisms, such as N_2_-fixers or mycorrhizal fungi [64]. As a result, consecutive monoculture might lead to an alteration of soil microbial community, and an accumulation of some rhizosphere-inhabiting microbes that were known to participate in detrimental interactions with the plant, and PGPRS population decline [65].

Most of the studies on *R. glutinosa* in the past have focused on the plant's autotoxicity. It was suggested that the undesirable effects brought about by consecutive monoculture stemmed from the autotoxicity by the root exudates [3]. However, the exact mechanism of the autotoxicity remains unclear. Some argued that the root exudates or litter could affect the rhizospheric microorganisms. For instance, stachyose, a major component in *R. glutinosa* tuber, significantly affects the microbial equilibrium in the rhizosphere [32]. The root exudates might cancel out soil bacteriostasis, and selectively promote the growth of specific microbes. In turn, the dominant microflora induce changes on plant's metabolisms, and increase exudates secretion [66], [67]. Similarly, our metaproteomic analysis also showed that all proteins involved in the carbohydrate/energy, amino acid and secondary metabolisms were up-regulated, as compared to the control, in both one- and two-year consecutive monoculture soils. Plant roots release organic compounds (e.g., stachyose) based on needs of its C and N metabolism. Most of these pathways participated in the soil nutrient cycles, including C and N cycling [20]. Accumulation of root exudates in the rhizosphere after *R. glutinosa* cultivation can rapidly cause pathogen proliferation in the replanted soils [68]. Under consecutive monoculture, the root exudates residue and the luxuriant pathogen growth could retard *R. glutinosa* development from its seeding to elongating stage, resulting in significantly declines on yield and quality [7], [68]. In other words, the microorganisms directly affected the quantity and composition of the root exudates, which inevitably altered the rhizosphere. In addition, changes in the microbial composition might also affect the nutrient cycling in soil, and therefore, the plant nutrition, which was shown by the changes in the root activity and N and K contents of the *R. glutinosa* plants (Table 2, 3). Our field experiment showed the typical effect of the consecutive monoculture on *R. glutinosa* (Table 1). The results showed that the root activity of the two-year consecutive monocultured plants was significantly lower than that of the one-year monocultured plants (*P* ≤ 0.05). The K content in the bled sap was significantly lower for the two-year than the one-year monocultured *R. glutinosa*. It is suggested that there might be some growth inhibitory factors that affected the plant's nutrient absorption in the ecosystem. The N and K contents, especially K, of the two-year monocultured *R*. *glutinosa* were significantly lower than those of the one-year counterparts.

Our soil metaproteomic analysis results provide a new insight into the biological functions of soil proteins, and a solid foundation to understand the interactions between the microorganisms and plants in the soil ecosystem. However, it should be noted that the databases for soil protein identification are still incomplete. An improved availability of protein database derived from environmental samples and *de novo* sequencing strategies will undoubtedly facilitate protein identification, environmental metaproteomic analysis and functional interpretation.

## Materials and Methods

### Soil samples

The *R. glutinosa* cultivar, ‘Wen 85-5’, was kindly provided by Wen Agricultural Institute, Jiaozuo, Henan, China for the study conducted on plots located at the Sunshine Agricultural Demonstration District, Jiaozuo, Henan (35°19′N, 113°51′E). The location was believed to be the most desirable natural setting for the growth of *R. glutinosa*. In the area used for planting wheat as the former crop, 3 random plots (12 m^2^) for each of the 3 cultivation patterns were designed for the trial test. The cultivation pattern included fallow plots as the control (CK) and two treatments, i.e., one-year monoculture (NP) and two-year consecutive monoculture (CM). Individual *R. glutinosa* tubers (3–4 cm in length) were planted on the plots with a spacing at 25×30 cm among plants. The plantings for the two-year consecutive monoculture were made on April 15, 2008 and April 15, 2009, and on April 15, 2009 for the one-year treatment.

Fifty days after planting, 10 plants from the two treatments were randomly excavated every ten days for samples. The sampled plants were carefully washed for the dry matter determination of both above- and below-ground biomasses in a 70°C oven for drying until constant weight. The R/S ratio was calculated by using the formula: R/S =  dry weight of tubers and roots/dry weight of shoots. On the 70th, 95th and 110th day after planting, 10 plants from the treatment groups were blanched in an oven at 105°C for 20 min, and dried at 70°C until constant weight. The dried samples were also used for N, P and K determinations [69].

Soil samples were obtained from 5 random locations on each plot at the tuber formation stage on November 30, 2009. The plants were carefully uprooted with a forked spade. Their roots were shaken to remove loosely attached soil. The rhizosphere soil samples were stored at −80°C for the determination of total phenolic acids content. For protein extraction, the soil samples were dried at 70°C for 2 h, pulverized in a mortar, and sieved through a 2 mm mesh to facilitate the process.

### Determination of root activity

Root activity was determined by BI of the plant [70]. On the 95th, 110th and 135th day after planting, 10 plants from each treatment group were randomly selected. Each plant was cut on the stem 2–3 cm above the ground at 5 p.m. The cut on the stem that remained with the root system was capped with a tube filled with sufficient absorbent cotton to collect the bleeding sap. At 8 a.m. the next morning, the tube was removed and weighted as *W2*. The time interval for the sap sampling was *T* = 15 h. The root activity was represented as BI  =  (*W2- W1*) /*T*, where, *W1*  =  weight of tube and cotton prior to sap collection. The K content in the bled sap was also determined.

### Determination of total phenolic acids in rhizospheric soil

Total phenolic acid content in rhizospheric soil was measured by the phosphomolybdic-phosphotungstic acid phenol reagent colorimetry. Briefly, 3 g of a soil sample were mixed with 30 ml deionized water (pH 4.0) by shaking at 200 rpm at ambient temperature for 1 h. The mixture was centrifuged at 250 g for 15 min, and filtered with filter paper. In 3.25 ml of the filtrate (i.e., PAE), 0.25 ml phenol reagent and 1.25 ml of 1 M sodium carbonate were added with shaking. After incubation at ambient temperature for 40 min, the optical absorption of the mixture was obtained at 770 nm. Three replications were used for each treatment. The total phenolic acid content in the soil samples was calculated by comparison with their respective standard curves. In addition, the filtrate was used to determine the effects of PAE on the length of lettuce root. The average root length (RL) was obtained according to the following procedure. Four ml PAE were placed in a 250 ml beaker with a filter paper. Ten pre-germinated lettuce (*Lactuca sativa* L.) seeds were put in each of the 5 beakers for each treatment. The lettuce plants were incubated at 25°C for 5 d (12 h in darkness, 12 h under light per day). Length of the lettuce roots was measured. The root growth inhibition rate (IR) was calculated by using the formula: IR =  (average RL of control plants – average RL of treatment plants / average RL of control plants) ×100%.

The phenol reagent used in this study was prepared as follows. Twelve g sodium tungstate, 5 g sodium molybdate, 70 ml deionized water, 4 ml phosphoric acid (85%), and 12 ml hydrochloric acid (36.7%) were put into a 250 ml matrass and heated in boiling water for 1 h. Then 6 g aluminum sulfate and 0.4 ml bromine water were added into the mixture, and heated in boiling water for 25 min. After coagulation, the mixture was diluted to 100 ml with deionized water and filtered with filter paper. The filtrate was diluted with equal volume of deionized water and stored at 4°C in preparation for use.

### Protein extraction and purification

In order to apply the metaproteomic approach for the study, it is critical that a high resolution and sensitivity on the 2-DE be achieved. The soil proteins from cultivated samples were extracted and purified by the following protocol developed in our lab [71]. Briefly, 1 g of dry cultivated soil powder were extracted using 5 mL of 0.05 M citrate buffer (pH 8.0) and 5 mL of 1.25% SDS buffer (1.25% w/v SDS, 0.1 M Tris-HCl, pH 6.8, 20 mM DTT), respectively. Then the proteins obtained from both citrate extraction and SDS extraction were solubilized and mixed in the same rehydration solution for SDS-PAGE and 2D-polyacrylamide gel electrophoresis (2-DE). Prior to electrophoresis, protein concentration was determined by Bradford assay using dilutions of bovine serum albumin as standards.

### SDS-PAGE and 2D-PAGE of extracted proteins

The extracted proteins were separated by SDS-PAGE [72] and 2D-polyacrylamide gel electrophoresis [73]. To prepare for the electrophoresis, protein pellets were first dissolved in an appropriate lysis solution (7 M urea, 2 M thiourea, 65 mM DTT and 4% CHAPS) with sonication for 10 min, and followed by centrifugation at 18,000 g and ambient temperature for 15 min to obtain the supernatant for protein separation.

For SDS-PAGE, the solubilized proteins were mixed with 5× loading buffer (60 mM Tris–HCl, pH 6.8, 25% glycerol, 2% SDS, 14.4 mM β-mercaptoethanol and 0.1% bromophenol blue), and incubated at 95°C for 5 min. Discontinuous SDS-PAGE was performed using a 5% stacking gel and a 10% separating gel. The stacking gel was connected to a constant 8 mA current, and the separating gel, 15 mA current. Unstained protein molecular weight (MW) marker (14.4–116 kD) was loaded. After the electrophoresis, with gentle shaking at ambient temperature, gels were stained for 2 h with 0.03% (w/v) Coomassie Brilliant Blue R-250, 50% methanol and 10% glacial acetic acid. Subsequently, the gels were de-stained several times with 5% methanol and 7% glacial acetic acid with gentle shaking.

For the 2-DE, a 150 μg soil protein sample was loaded. An isoelectric focusing (IEF) tube gel (17 cm ×0.02 cm) containing 8 M urea, 3.5% acrylamide, 2% NP-40, 2% ampholines (GE Healthcare, Uppsala, Sweden)(ratio of pH 3.5–10.0 to pH 5.0–8.0 was 1∶5 for a nonlinear gel) was prepared. The samples were separated by IEF in the first dimension, and SDS-PAGE using a 5% stacking gel and a 10% separating gel in the second dimension. MW markers ranging from 14.4 to 116 kDa (Promega, Madison, USA) were used in the second dimension for size standardization. After the electrophoresis, gels were stained with silver nitrate [74], scanned with Imagescan, and analyzed with the ImageMaster™ 2D Platinum software 5.0 (GE Healthcare, Uppsala, Sweden). Repeatability analysis of 2-DE maps of soil proteins was carried out through scatter plots with ImageMaster™ 2D Platinum software 5.0 (GE Healthcare, Uppsala, Sweden) according to the manufacture's instructions. Protein spots with greater than 1.5-fold change from the normalized volume were considered differentially expressed.

### Protein identification by LIFT-MALDI TOF-TOF MS

It is most feasible to identify proteins with the available metagenomic sequences. However, short of the environmental sequence data, proteins obtained from environmental samples can also be identified reliably from their respective *de novo* peptide sequences by searching against the current databases using the MS basic local alignment search tool (BLAST) algorithm [75].

Protein spots of interest are excised manually from gels for mass spectrometric analysis [76]. In this study, each selected gel spot was rinsed twice with deionized water, de-stained with 25 mM ammonium bicarbonate in water/acetonitrile (50/50) solution, and treated with 1∶1 solution of 30 mM potassium ferricyanide and 100 mM sodium thiosulfate and then equilibrated in 50 mM ammonium bicarbonate (pH 8). After dehydrating with acetonitrile and drying in a Speed-Vac centrifuge (Thermo Fisher Scientific, Waltham, MA), the gel spot was rehydrated in a minimal volume of trypsin (Promega, Madison, USA) solution (12.5 μg/ml in 25 mM ammonium bicarbonate) and incubated at 37°C overnight. The liquid was transferred to a 200 μl micro-centrifuge tube, while the precipitated solids extracted once with the buffer (67% acetonitrile containing 2.5% trifluoroacetic acid). Then, both liquids were combined and completely dried in a SpeedVac centrifuge prior to re-suspension in 5 μl of 0.1% trifluoroacetic acid followed by mixing in 1∶1 ratio with a saturated solution of α-cyano-4-hydroxy-trans-cinnamic acid in 30% acetonitrile containing 0.1% trifluoroacetic acid.

One μl of the abovementioned solution were spotted onto stainless steel sample target plates. Peptide mass spectra were obtained on a Bruker UltraFlex III MALDI TOF/TOF mass spectrometer (Bruker Daltonics, Karlsruhe, Germany). Data were acquired in the positive MS reflector mode using 6 external standards for the instrument calibration (Peptide Calibration Standard II, Bruker Daltonics). Mass spectra were obtained for each sampled spot by accumulation of 600–800 laser shots in an 800–5,000 Da mass range. For the MS/MS spectra, 5 most abundant precursor ions per sample were selected for subsequent fragmentation, and 1,000–1,200 Da laser shots were accumulated per precursor ion. The criterion for precursor selection was a minimum S/N of 50.

### Database search

Both MS and MS/MS data were interpreted and processed by using Flexanalysis 3.0 (Bruker Daltonics). The obtained MS and MS/MS spectra per spot were combined, and submitted to MASCOT search engine (V2.3, Matrix Science, London, U.K.) by Biotools 3.1 (Bruker Daltonics). Parameters selected included: the NCBInr database (2010.01.20, 10348164 sequences; 3529470745 residues) in SwissProt (http://www.matrixscience.com/search_form_select.html), taxonomy of all entries followed by ‘Bacteria’ or ‘Fungi’ database, trypsin of the digestion enzyme, up to one missed cleavage site, parent ion mass tolerance at 100 ppm, MS/MS mass tolerance of 0.6 Da, carbamidomethylation of cysteine (global modification), and methionine oxidation (variable modification). The probability score (95% confidence level) calculated by the software was used as a criterion for correct identification.

Due to the vast varieties of soil sample sources, the protein mass spectra were searched sequentially against three databases (all entries, ‘Bacteria’ and ‘Fungi’ database). Firstly, the item, ‘all entries’, was entered for the search. Then, the ‘Bacteria’ or ‘Fungi’ database, was separately applied when significant matching was not obtained in the first attempt. The above strategy alleviated the problem of missing some of the mass spectra for matches in searching against ‘all entries’, and allowed significant matching results by searching against ‘Bacteria’ and ‘Fungi’ databases. Both MS/MS and MS data were utilized for the identification of proteins. The proteins sharing equal searching by MS/MS and MS were preferentially selected. Then, proteins matched at least two MS/MS peptides or three PMFs were subjected to further identification. Only the proteins with the highest score and similar predicted molecular mass were selected. Finally, these unknown proteins were identified by using mass spectrometry based BLAST (MS BLAST) [77]–[79].

## Supporting Information

Figure S1 **Proposed metabolic model for rhizosphere soil proteins as inferred by metaproteomic data.** Identification numbers (E.C.-.-.-.-.) refer to identified proteins. EMP: Embden-Meyerhof pathway. TCA: tricarboxylic acid cycle. GAC: glyoxylic acid cycle. PPP: pentose phosphate pathway.
(DOC)

Table S1 Proteins identified by equal MS/MS and MS searching.
(DOC)

Table S2 Proteins identified by MS/MS.
(DOC)

Table S3 Proteins identified by MS.
(DOC)

Table S4 Differentially expressed proteins identified by MS/MS.
(DOC)
