# Supplementary material for: Comparative Metaproteomic Analysis on Consecutively Rehmannia glutinosa-Monocultured Rhizosphere Soil
Source: PLoS One. 2011 May 31;6(5):e20611. doi: 10.1371/journal.pone.0020611 (PMC3105091; doi:10.1371/journal.pone.0020611)
Supplement: Figure S1 — Proposed metabolic model for rhizosphere soil proteins as inferred by metaproteomic data. Identification numbers (E.C.-.-.-.-.) refer to identified proteins. EMP: Embden-Meyerhof pathway. TCA: tricarboxylic acid cycle. GAC: glyoxylic acid cycle. PPP: pentose phosphate pathway. (DOC) [file pone.0020611.s001.doc]

**
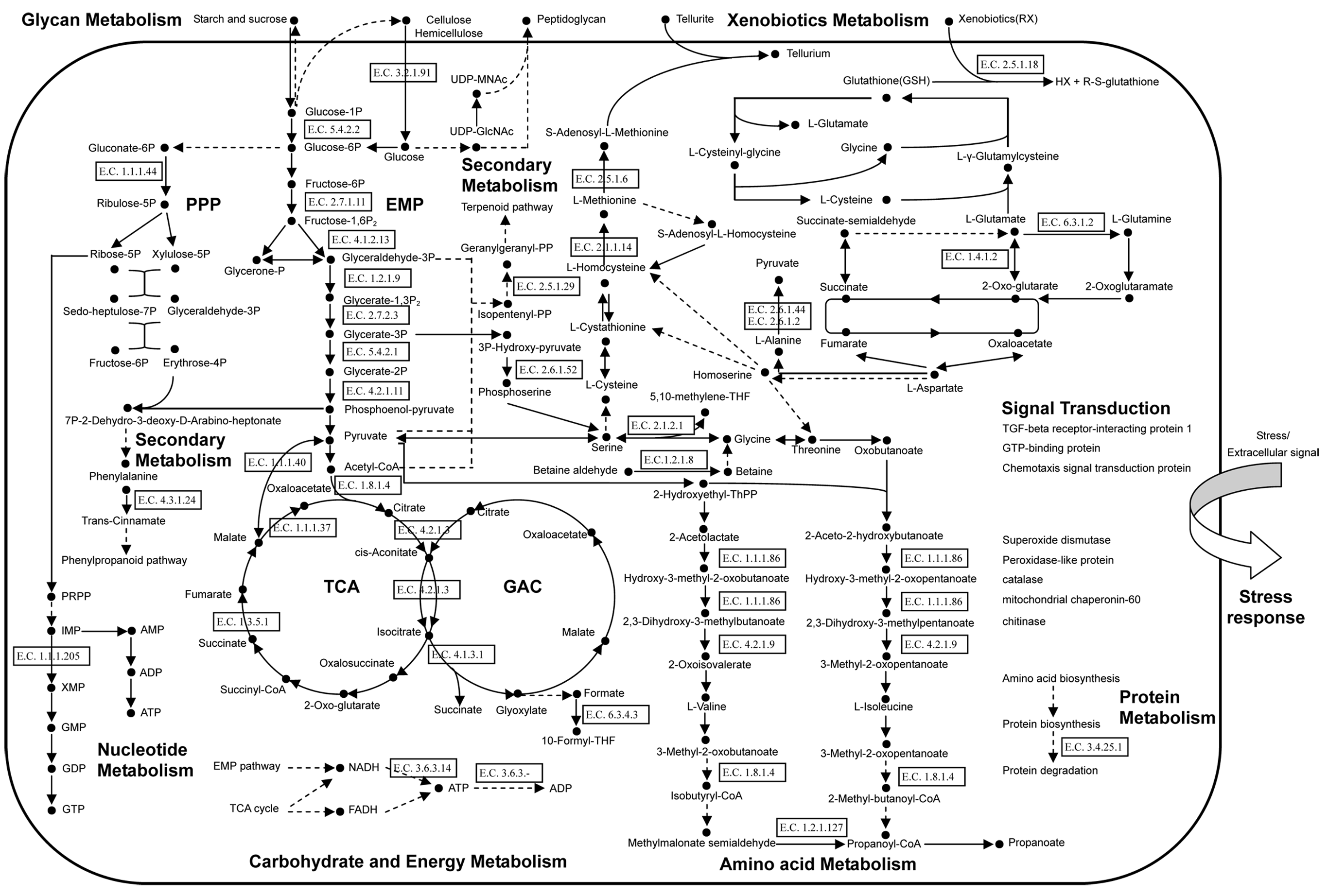
**

**Figure S1. Proposed metabolic model for rhizosphere soil proteins as inferred by metaproteomic data.** Identification numbers (E.C.-.-.-.-.) refer to identified proteins. EMP: Embden-Meyerhof pathway. TCA: tricarboxylic acid cycle. GAC: glyoxylic acid cycle. PPP: pentose phosphate pathway.
