# Supplementary material for: Comparative Metaproteomic Analysis on Consecutively Rehmannia glutinosa-Monocultured Rhizosphere Soil
Source: PLoS One. 2011 May 31;6(5):e20611. doi: 10.1371/journal.pone.0020611 (PMC3105091; doi:10.1371/journal.pone.0020611)
Supplement: Table S1 — Proteins identified by equal MS/MS and MS searching. (DOC) [file pone.0020611.s002.doc]

**Table S1.** Proteins identified by equal MS/MS and MS searching.

| Spot no. ^a)^ | GI no.^b)^ | Protein name (Identification number ^c)^) | Score  (PMF) ^d)^ | PMF/  converage ^e)^ | MW/p*I*^f)^ | Score  (MS-MS) ^g)^ | Pept ^h)^ | Species | Database ^i)^ | Function |
| --- | --- | --- | --- | --- | --- | --- | --- | --- | --- | --- |
| 2 | [gi|115450595](http://www.matrixscience.com/cgi/protein_view.pl?file=../data/20100121/FtmponsOh.dat&hit=2) | Aconitate hydratase (E.C. 4.2.1.3) | 146 | 25/32% | 106862/6.45 | 239 | 4 | *Oryza sativa* | All entries | TCA, GAC ^1^ |
| 3 | [gi|162461914](http://www.matrixscience.com/cgi/protein_view.pl?file=../data/20100118/FtmpffaOE.dat&hit=gi|162461914&px=1&ave_thresh=53&_sigthreshold=0.05&_server_mudpit_switch=0.001) | Phenylalanine ammonia-lyase (E.C. 4.3.1.24) | 78 | 15/24% | 75336/6.52 | 269 | 3 | *Zea mays* | All entries | Secondary metabolism ^2^ |
| 4 | gi|108862992 | 5-methyltetrahydropteroyltriglutamate-homocysteine methyltransferase (E.C. 2.1.1.14) | 120 | 16/25% | 84925/5.93 | 437 | 5 | *Oryza sativa* | All entries | Amino acid metabolism ^3^ |
| 6 | [gi|110288669](http://www.matrixscience.com/cgi/protein_view.pl?file=../data/20100118/Ftmpffctt.dat&hit=3) | Putative enolase (E.C. 4.2.1.11) | 215 | 15/61% | 51834/5.84 | 353 | 2 | *Oryza sativa* | All entries | EMP ^4^ |
| 7 | [gi|115470967](http://www.matrixscience.com/cgi/protein_view.pl?file=../data/20100118/FtmpffcOt.dat&hit=2) | Methylmalonate-semialdehyde dehydrogenase[acylating] (E.C. 1.2.1.127) | 92 | 23/53% | 57666/5.98 | 393 | 4 | *Oryza sativa* | All entries | Amino acid metabolism ^5^ |
| 8 | gi|115467370 | Pyrophosphate-dependent 6-phosphofructose-1-kinase (E.C. 2.7.1.11) | 95 | 15/34% | 61907/6.01 | 166 | 3 | *Oryza sativa* | All entries | EMP ^6^ |
| 9 | [gi|115459078](http://www.matrixscience.com/cgi/protein_view.pl?file=../data/20100106/FtmmSxene.dat&hit=gi|115459078&px=1&ave_thresh=53&_sigthreshold=0.05&_server_mudpit_switch=0.001) | Glyceraldehyde-3-phosphate dehydrogenase (E.C. 1.2.1.9) | 164 | 20/64% | 36921/6.34 | 369 | 3 | *Oryza sativa* | All entries | EMP ^7^ |
| 10 | [gi|3024122](http://www.matrixscience.com/cgi/protein_view.pl?file=../data/20100121/FtmponTmE.dat&hit=gi|3024122&px=1&ave_thresh=53&_sigthreshold=0.05&_server_mudpit_switch=0.001) | S-adenosylmethionine synthetase (E.C. 2.5.1.6) | 131 | 17/52% | 43330/5.68 | 392 | 4 | *Oryza sativa* | All entries | Amino acid metabolism ^8^ |
| 11 | gi|51536102 | Putative formate--tetrahydrofolate ligase (E.C. 6.3.4.3) | 86 | 15/25% | 68639/6.55 | 122 | 3 | *Oryza sativa* | All entries | One carbon pool ^9^ |
| 12 | [gi|115455349](http://www.matrixscience.com/cgi/protein_view.pl?file=../data/20100118/FtmpffuOO.dat&hit=gi|115455349&px=1&ave_thresh=53&_sigthreshold=0.05&_server_mudpit_switch=0.001) | Exoglucanase precursor (E.C. 3.2.1.91) | 125 | 19/36% | 68191/7.23 | 189 | 3 | *Oryza sativa* | All entries | Glycan metabolism ^10^ |
| 13 | [gi|115485405](http://www.matrixscience.com/cgi/protein_view.pl?file=../data/20100121/FtmponsTO.dat&hit=2) | Serine hydroxymethyltransferase (E.C. 2.1.2.1) | 78 | 18/36% | 51799/7.16 | 130 | 1 | *Oryza sativa* | All entries | Amino acid metabolism ^11^ |
| 14 | gi|115450567 | Glutathione S-transferase GSTF15 (E.C. 2.5.1.18) | 89 | 7/23% | 25755/6.67 | 255 | 4 | *Oryza sativa* | All entries | Xenobiotics Metabolism ^12^, Amino acid metabolism ^13^ |
| 23 | gi|54606800 | NADP dependent malic enzyme (E.C. 1.1.1.40) | 172 | 25/38% | 65824/5.79 | 346 | 4 | *Oryza sativa* | All entries | Pyruvate metabolism ^14^ |
| 26 | [gi|115440691](http://www.matrixscience.com/cgi/protein_view.pl?file=../data/20100118/FtmpffTET.dat&hit=gi|115440691&px=1&ave_thresh=53&_sigthreshold=0.05&_server_mudpit_switch=0.001) | 2,3-bisphosphoglycerate-independent phosphoglycerate mutase (E.C. 5.4.2.1) | 213 | 27/49% | 60980/5.42 | 495 | 5 | *Oryza sativa* | All entries | EMP ^15^ |
| 29 | gi|115436818 | Thiamine pyrophosphate enzyme | 170 | 22/45% | 61187/5.95 | 321 | 4 | *Oryza sativa* | All entries | EMP, TCA ^16^ |
| 30 | gi|115470493 | Succinate dehydrogenase [ubiquinone] flavoprotein subunit (E.C. 1.3.5.1) | 84 | 16/26% | 69494/6.61 | 125 | 3 | *Oryza sativa* | All entries | TCA ^17^ |
| 32 | [gi|115435028](http://www.matrixscience.com/cgi/protein_view.pl?file=../data/20100121/FtmponeeO.dat&hit=gi|115435028&px=1&ave_thresh=53&_sigthreshold=0.05&_server_mudpit_switch=0.001) | Mitochondrial processing peptidase (E.C. 3.4.24.64) | 121 | 15/39% | 54141/6.65 | 233 | 3 | *Oryza sativa* | All entries | Protein metabolism ^18^ |
| 34 | gi|115451029 | Similar to Alanine:glyoxylate aminotransferase-like protein (E.C. 2.6.1.44) | 109 | 18/32% | 53089/8.49 | 71 | 1 | *Oryza sativa* | All entries | Amino acid metabolism ^19^ |
| 40 | gi|222625763 | Glycine hydroxymethyltransferase (E.C. 2.1.2.1) | 160 | 25/49% | 53764/8.46 | 98 | 2 | *Oryza sativa* | All entries | Amino acid metabolism ^20^ |
| 42 | gi|91204063 | Integron integrase | 105 | 15/37% | 50230/9.34 | 144 | 3 | *Oryza sativa* | All entries | Genetic information processing ^21^ |
| 46 | gi|115470967 | Methylmalonate-semialdehyde dehydrogenase (E.C. 1.2.1.127) | 109 | 22/46% | 57666/5.98 | 379 | 4 | *Oryza sativa* | All entries | Amino acid metabolism ^5^ |
| 54 | gi|115465974 | Cytosolic 6-phosphogluconate dehydrogenase (E.C. 1.1.1.44) | 109 | 13/37% | 52973/5.85 | 165 | 2 | *Oryza sativa* | All entries | PPP ^22^ |
| 65 | gi|115455879 | Similar to Glutamate dehydrogenase (E.C. 1.4.1.2) | 115 | 14/42% | 44599/6.15 | 168 | 3 | *Oryza sativa* | All entries | Amino acid metabolism ^23^ |
| 75 | gi|115448577 | Pyruvate dehydrogenase E1 component alpha subunit (E.C. 1.2.4.1) | 97 | 12/28% | 43017/7.64 | 193 | 4 | *Oryza sativa* | All entries | EMP ^24^ |
| 103 | gi|51090388 | Putative PrMC3 | 116 | 12/43% | 34540/5.61 | 367 | 3 | *Oryza sativa* | All entries | Stress/defense response ^25, 26^ |
| 105 | gi|51090388 | Putative PrMC3 | 107 | 16/59% | 34540/5.61 | 296 | 3 | *Oryza sativa* | All entries | Stress/defense response ^25, 26^ |
| 116 | gi|115475824 | Similar to TGF-beta receptor-interacting protein 1 | 90 | 11/33% | 36527/5.94 | 200 | 3 | *Oryza sativa* | All entries | Signal transduction ^27^ |
| 125 | [gi|121333](http://www.matrixscience.com/cgi/protein_view.pl?file=../data/20100106/FtmmSxEOL.dat&hit=gi|121333&px=1&ave_thresh=52&_sigthreshold=0.05&_server_mudpit_switch=0.001) | Glutamine synthetase root isozyme A (E.C. 6.3.1.2) | 122 | 15/53% | 39435/6.12 | 122 | 3 | *Oryza sativa* | All entries | Amino acid metabolism ^28^ |
| 127 | gi|968996 | Glyceraldehyde-3-phosphate dehydrogenase (E.C. 1.2.1.9) | 139 | 14/50% | 36641/6.61 | 379 | 3 | *Oryza sativa* | All entries | EMP ^7^ |
| 129 | [gi|968996](http://www.matrixscience.com/cgi/protein_view.pl?file=../data/20100106/FtmmSxYeT.dat&hit=gi|968996&px=1&ave_thresh=53&_sigthreshold=0.05&_server_mudpit_switch=0.001) | Glyceraldehyde-3-phosphate dehydrogenase (E.C. 1.2.1.9) | 161 | 16/54% | 36641/6.61 | 594 | 5 | *Oryza sativa* | All entries | EMP ^7^ |
| 130 | [gi|78099751](http://www.matrixscience.com/cgi/protein_view.pl?file=../data/20100121/FtmponTOt.dat&hit=gi|78099751&px=1&ave_thresh=53&_sigthreshold=0.05&_server_mudpit_switch=0.001) | Fructose-bisphosphate aldolase cytoplasmic isozyme (E.C. 4.1.2.13) | 100 | 10/38% | 39238/6.96 | 404 | 4 | *Oryza sativa* | All entries | EMP ^29^ |
| 140 | [gi|108706511](http://www.matrixscience.com/cgi/master_results.pl?file=../data/20100106/FtmmSxTSO.dat#Hit1) | Proteasome subunit alpha type 6 (E.C. 3.4.25.1) | 130 | 13/43% | 32472/7.05 | 196 | 3 | *Oryza sativa* | All entries | Protein metabolism ^30^ |
| 143 | gi|115480019 | Proteasome subunit beta type 1 (E.C. 3.4.25.1) | 136 | 11/50% | 24608/6.43 | 92 | 2 | *Oryza sativa* | All entries | Protein metabolism ^30^ |
| 148 | gi|115448935 | Beta 1 subunit of 20S proteasome (E.C. 3.4.25.1) | 109 | 12/47% | 26371/5.47 | 264 | 3 | *Oryza sativa* | All entries | Protein metabolism ^31^ |

Note: ^a)^ The numbering corresponds to the 2-DE gel in figure 4. ^b)^ GI number in NCBI. ^c)^ a unique 4-digit identification number for enzyme identification by the Enzyme Commission (E.C.). ^d)^ MASCOT score of PMF. ^e)^ The number of peptides identified by MS/sequence percentage coverage. ^f)^ Theoretical molecular weight and p*I*. ^g)^ MASCOT score of MS/MS. ^h)^ Number of peptides identified by MS/MS. ^i)^ The used database in the process of MASCOT search. EMP: Embden-Meyerhof pathway. TCA: tricarboxylic acid cycle. GAC: glyoxylic acid cycle. PPP: pentose phosphate pathway.

**Reference**

1. Beinert H, Kennedy MC (1993) Aconitase, a two-faced protein: enzyme and iron regulatory factor. Faseb J 7: 1442-1449.
2. Elkind [Y](http://www.pnas.org/search?author1=Y+Elkind&sortspec=date&submit=Submit), Edwards [R](http://www.pnas.org/search?author1=R+Edwards&sortspec=date&submit=Submit), Mavandad [M](http://www.pnas.org/search?author1=M+Mavandad&sortspec=date&submit=Submit), Hedrick [SA](http://www.pnas.org/search?author1=S+A+Hedrick&sortspec=date&submit=Submit), Ribak [O](http://www.pnas.org/search?author1=O+Ribak&sortspec=date&submit=Submit), et al. (1990) Abnormal plant development and down-regulation of phenylpropanoid biosynthesis in transgenic tobacco containing a heterologous phenylalanine ammonia-lyase gene. P Natl Acad Sci USA 87: 9057-9061.
3. Whitfield CD, Steers EJ Jr, Weisbach H (1970) Purification and properties of 5-methyltetrahydropteroyltriglutamate-homocysteine transmethylase. J Biol Chem 245: 390-401.
4. Reed GH, Poyner RR, Larsen TM, Wedekind JE, Rayment I (1996) Structural and mechanistic studies of enolase. Curr Opin Struct Biol 6: 736-743.
5. Naoki T, Hideyuki T, Hidemi K, Makoto M, Shoichiro A, et al. (2005) Proteome approach to characterize the methylmalonate-semialdehyde dehydrogenase that is regulated by gibberellin. J Proteome Res 4: 1575-1582.
6. Suzuki J, Mutton MA, Ferro MI, Lemos MV, Pizauro JM, et al. (2003) Putative pyrophosphate phosphofructose 1-kinase genes identified in sugar cane may be getting energy from pyrophosphate. Genet Mol Res 2: 376-382.
7. Michael AS (1999) New insights into an old protein: the functional diversity of mammalian glyceraldehyde-3-phosphate dehydrogenase. BBA-Biomembranes 1432: 159-184.
8. Frank VB, Rudy D, Jan G, Marc VM, Allan C (1994) Characterization of a s-adenosylmethionine synthetase gene in rice. Plant Physiol 105: 1463-1464.
9. Marx CJ, Laukel M, Vorholt JA, Lidstrom ME (2003) Purification of the formate-tetrahydrofolate ligase from methylobacterium extorquens AM1 and demonstration of its requirement for methylotrophic growth. J Bacteriol 185: 7169-7175.
10. Han Y, Chen H (2010) Biochemical characterization of a maize stover beta-exoglucanase and its use in lignocellulose conversion. Bioresour Technol 101: 6111-6117.
11. Ogawa H, Gomi T, Fujioka M (2000) Serine hydroxymethyltransferase and threonine aldolase: are they identical? Int J Biochem Cell Biol 32: 289-301.
12. Cho HY, Kong KH (2005) Molecular cloning, expression, and characterization of a phi-type glutathione S-transferase from *Oryza sativa*. Pestic Biochem Phys 83: 29-36.
13. Udomsinprasert R, Pongjaroenkit S, Wongsantichon J, Oakley AJ, Prapanthadara LA, et al. (2005) Identification, characterization and structure of a new Delta class glutathione transferase isoenzyme. Biochem J 388: 763-771.
14. Rothermel BA, Nelson T (1989) Primary structure of the maize NADP-dependent malic enzyme. J Biol Chem, 264: 19587-19592.
15. Chevalier N, Rigden DJ, Van Roy J, Opperdoes FR, Michels PA (2000) *Trypanosoma brucei* contains a 2,3-bisphosphoglycerate independent phosphoglycerate mutase. Eur J Biochem 267: 1464-1472
16. Arjunan P, Umland T, Dyda F, Swaminathan S, Furey W, et al. (1996) Crystal structure of the thiamin diphosphate-dependent enzyme pyruvate decarboxylase from the yeast *Saccharomyces cerevisiae* at 2.3 A resolution. J Mol Biol 256: 590-600.
17. Oyedotun KS, Lemire BD (2004) The quaternary structure of the Saccharomyces cerevisiae succinate dehydrogenase. Homology modeling, cofactor docking, and molecular dynamics simulation studies. J Biol Chem 279: 9424-9431.
18. Luciano P, Géli V (1996) The mitochondrial processing peptidase: function and specificity. Experientia 52: 1077-1082.
19. Noguchi T, Okuno E, Takada Y, Minatogawa Y, Okai K, et al. (1978) Characteristics of hepatic alanine-glyoxylate aminotransferase in different mammalian species. Biochem J 169: 113-122.
20. Blakley RL (1960) A spectrophotometric study of the reaction catalysed by serine transhydroxymethylase. Biochem J 77: 459-465.
21. Messier N, Roy PH (2001) Integron integrases possess a unique additional domain necessary for activity. J Bacteriol 183: 6699-6706.
22. Bailey-Serres J, Nguyen MT (1992) Purification and characterization of cytosolic 6-phosphogluconate dehydrogenase isozymes from maize. Plant Physiol 100: 1580-1583.
23. Mungur R, Glass AD, Goodenow DB, Lightfoot DA (2005) Metabolite fingerprinting in transgenic *Nicotiana tabacum* altered by the *Escherichia coli* glutamate dehydrogenase gene. J Biomed Biotechnol: 198-214.
24. Arjunan P, Nemeria N, Brunskill A, Chandrasekhar K, Sax M, et al. (2002) Structure of the pyruvate dehydrogenase multienzyme complex E1 component from *Escherichia coli* at 1.85 A resolution. Biochemistry 41: 5213-5221.
25. Lee DG, Ahsan N, Lee SH, Lee JJ, Bahk JD, et al. (2009) Chilling stress-induced proteomic changes in rice roots. J Plant Physiol 166: 1-11.
26. Walden AR, Walter C, Gardner RC (1999) Genes expressed in *pinus radiata* male cones include homologs to anther-specific and pathogenesis response genes. Plant Physiol 121: 1103-1116.
27. Hu X, Zhou H, Hu F, Xu J, Zhao Y, et al. (2008) Recognition and characterization of TGF-beta receptor interacting protein 1 (TRIP-1) containing WD40 repeats from Clonorchis sinensis by bioinformatics, cloning, and expression in *Escherichia coli*. Parasitol Res 103: 1151-1158.
28. Tingey SV, Walker EL, Coruzzi GM (1987) Glutamine synthetase genes of pea encode distinct polypeptides which are differentially expressed in leaves, roots and nodules. Embo J 6: 1-9.
29. Konishi H, Yamane H, Maeshima M, Komatsu S (2004) Characterization of fructose-bisphosphate aldolase regulated by gibberellin in roots of rice seedling. Plant Mol Biol 56: 839-848.
30. Suzanne E, Rayappa RG, Martin S, Christopher NL, David SL, et al. (2002) Proteasome subunit Rpn1 binds ubiquitin-like protein. Nat Cell Biol 4: 725-730.
31. Schmidtke G, Kraft R, Kostka S, Henklein P, Frömmel C et al. (1996) Analysis of mammalian 20S proteasome biogenesis: the maturation of beta-subunits is an ordered two-step mechanism involving autocatalysis. Embo J 15: 6887-6898.
