# Supplementary material for: Comparative Metaproteomic Analysis on Consecutively Rehmannia glutinosa-Monocultured Rhizosphere Soil
Source: PLoS One. 2011 May 31;6(5):e20611. doi: 10.1371/journal.pone.0020611 (PMC3105091; doi:10.1371/journal.pone.0020611)
Supplement: Table S2 — Proteins identified by MS/MS. (DOC) [file pone.0020611.s003.doc]

**Table S2.** Proteins identified by MS/MS.

| Spot no. ^a)^ | GI no. ^b)^ | Protein name (Identification number ^c)^) | Score ^d)^ | Pept ^e)^ | MW/p*I* ^f)^ | Species | Database ^g)^ | Function |
| --- | --- | --- | --- | --- | --- | --- | --- | --- |
| 1 | [gi|75225211](http://www.matrixscience.com/cgi/protein_view.pl?file=../data/20091207/FtmomnYeR.dat&hit=gi|75225211&px=1&ave_thresh=51&_sigthreshold=0.05&_server_mudpit_switch=0.001) | Putative aconitate hydratase (E.C. 4.2.1.3) | 136 | 2 | 98591/5.67 | *Oryza sativa* | All entries | TCA ^1^ |
| 5 | gi|115454931 | Similar to Phosphoglucomutase, cytoplasmic 2 (E.C. 5.4.2.2) | 85 | 2 | 63138/5.40 | *Oryza sativa* | All entries | EMP, PPP ^2^ |
| 15 | [gi|115477815](http://www.matrixscience.com/cgi/protein_view.pl?file=../data/20100121/FtmporTnO.dat&hit=gi|115477815&px=1&ave_thresh=53&_sigthreshold=0.05&_server_mudpit_switch=0.001) | Similar to Dihydroxy-acid dehydratase (E.C. 4.2.1.9) | 298 | 4 | 64237/7.12 | *Oryza sativa* | All entries | Amino acid metabolism ^3^ |
| 16 | [gi|115436320](http://www.matrixscience.com/cgi/protein_view.pl?file=../data/20091207/FtmomGaaO.dat&hit=gi|115436320&px=1&ave_thresh=52&_sigthreshold=0.05&_server_mudpit_switch=0.001) | Dihydrolipoamide dehydrogenase family protein (E.C. 1.8.1.4) | 69 | 2 | 53009/7.21 | *Oryza sativa* | All entries | Amino acid metabolism ^4^ |
| 17 | [gi|115436024](http://www.matrixscience.com/cgi/protein_view.pl?file=../data/20100118/FtmpffsSt.dat&hit=gi|115436024&px=1&ave_thresh=54&_sigthreshold=0.05&_server_mudpit_switch=0.001) | Superoxide dismutase [Mn] (E.C. 1.5.1.1) | 331 | 4 | 22634/7.77 | *Oryza sativa* | All entries | Stress/defense response ^5^ |
| 18 | [gi|125542289](http://www.matrixscience.com/cgi/protein_view.pl?file=../data/20100118/FtmpffunR.dat&hit=gi|125542289&px=1&ave_thresh=53&_sigthreshold=0.05&_server_mudpit_switch=0.001) | Glutathione S-transferase (E.C. 2.5.1.18) | 208 | 2 | 25575/6.72 | *Oryza sativa* | All entries | Xenobiotics Metabolism ^6^, Amino acid metabolism ^7^ |
| 22 | [gi|108862990](http://www.matrixscience.com/cgi/protein_view.pl?file=../data/20091207/FtmomnSST.dat&hit=gi|108862990&px=1&ave_thresh=51&_sigthreshold=0.05&_server_mudpit_switch=0.001) | 5-methyltetrahydropteroyltriglutamate-homocysteine methyltransferase (E.C. 2.1.1.14) | 352 | 4 | 79272/7.19 | *Oryza sativa* | All entries | Amino acid metabolism ^8^ |
| 27 | [gi|115111257](http://www.matrixscience.com/cgi/protein_view.pl?file=../data/20091222/FtmmfaTeL.dat&hit=gi|115111257&px=1&ave_thresh=52&_sigthreshold=0.05&_server_mudpit_switch=0.001) | Betaine aldehyde dehydrogenase (E.C. 1.2.1.8) | 188 | 2 | 55361/5.29 | *Oryza sativa* | All entries | Amino acid metabolism ^9^ |
| 33 | gi|3868754 | Catalase (E.C. 1.11.1.6) | 147 | 2 | 57052/6.49 | *Oryza sativa* | All entries | Stress/defense response ^10^ |
| 36 | gi|115465569 | Ketol-acid reductoisomerase (E.C. 1.1.1.86) | 256 | 4 | 62680/6.01 | *Oryza sativa* | All entries | Amino acid metabolism ^11^ |
| 39 | gi|115482382 | Mitochondrial chaperonin-60 | 204 | 4 | 61097/5.71 | *Oryza sativa* | All entries | Protein folding ^12^ |
| 41 | [gi|780372](http://www.matrixscience.com/cgi/protein_view.pl?file=../data/20091212/FtmolnctL.dat&hit=gi|780372&px=1&ave_thresh=52&_sigthreshold=0.05&_server_mudpit_switch=0.001) | Enolase (E.C. 4.2.1.11) | 386 | 5 | 48299/5.42 | *Oryza sativa* | All entries | EMP ^13^ |
| 47 | [gi|89280711](http://www.matrixscience.com/cgi/protein_view.pl?file=../data/20091222/FtmmfaTSO.dat&hit=gi|89280711&px=1&ave_thresh=53&_sigthreshold=0.05&_server_mudpit_switch=0.001) | ATP synthase F0 subunit 1 (E.C. 3.6.3.14) | 417 | 4 | 55532/5.85 | *Oryza sativa* | All entries | Oxidative phosphorylation ^14^ |
| 48 | [gi|89280711](http://www.matrixscience.com/cgi/protein_view.pl?file=../data/20091212/FtmolnaSe.dat&hit=gi|89280711&px=1&ave_thresh=53&_sigthreshold=0.05&_server_mudpit_switch=0.001) | ATP synthase F0 subunit 1 (E.C. 3.6.3.14) | 264 | 3 | 55532/5.85 | *Oryza sativa* | All entries | Oxidative phosphorylation ^14^ |
| 51 | [gi|12957707](http://www.matrixscience.com/cgi/protein_view.pl?file=../data/20091212/FtmolnanR.dat&hit=gi|12957707&px=1&ave_thresh=53&_sigthreshold=0.05&_server_mudpit_switch=0.001) | Putative inosine monophosphate dehydrogenase (E.C. 1.1.1.205) | 53 | 2 | 52851/6.03 | *Oryza sativa* | All entries | Nucleotide Metabolism ^15^ |
| 52 | [gi|11263](http://www.matrixscience.com/cgi/protein_view.pl?file=../data/20091222/FtmmfaTnt.dat&hit=gi|11263&px=1&ave_thresh=53&_sigthreshold=0.05&_server_mudpit_switch=0.001) | ATPase subunit (E.C. 3.6.3.-) | 110 | 2 | 55306/5.69 | *Beta vulgaris subsp* | All entries | Oxidative phosphorylation ^14^ |
| 58 | [gi|14018051](http://www.matrixscience.com/cgi/protein_view.pl?file=../data/20091212/FtmolnaOR.dat&hit=gi|14018051&px=1&ave_thresh=53&_sigthreshold=0.05&_server_mudpit_switch=0.001) | Putative alanine aminotransferase (E.C. 2.6.1.2) | 141 | 3 | 53229/6.23 | *Oryza sativa* | All entries | Amino acid metabolism ^16^ |
| 60 | [gi|3925239](http://www.matrixscience.com/cgi/protein_view.pl?file=../data/20100126/FtmpSiawT.dat&hit=gi|3925239&px=1&ave_thresh=53&_sigthreshold=0.05&_server_mudpit_switch=0.001) | 6-phosphogluconate dehydrogenase isoenzyme A (E.C. 1.1.1.44) | 85 | 2 | 19147/5.24 | *Zea mays* | All entries | PPP ^17^ |
| 66 | [gi|14018051](http://www.matrixscience.com/cgi/protein_view.pl?file=../data/20091212/Ftmolnawm.dat&hit=gi|14018051&px=1&ave_thresh=52&_sigthreshold=0.05&_server_mudpit_switch=0.001) | Putative alanine aminotransferase (E.C. 2.6.1.2) | 210 | 4 | 53229/6.23 | *Oryza sativa* | All entries | Amino acid metabolism ^16^ |
| 78 | [gi|38637314](http://www.matrixscience.com/cgi/protein_view.pl?file=../data/20091212/FtmolnatO.dat&hit=gi|38637314&px=1&ave_thresh=53&_sigthreshold=0.05&_server_mudpit_switch=0.001) | Putative GTP-binding protein | 170 | 3 | 44704/6.30 | *Oryza sativa* | All entries | Signal transduction ^18^ |
| 79 | [gi|115450835](http://www.matrixscience.com/cgi/protein_view.pl?file=../data/20091223/FtmmrnHSS.dat&hit=gi|115450835&px=1&ave_thresh=53&_sigthreshold=0.05&_server_mudpit_switch=0.001) | Phosphoserine aminotransferase, chloroplast precursor (E.C. 2.6.1.52) | 98 | 2 | 45302/8.53 | *Oryza sativa* | All entries | Amino acid metabolism ^19^ |
| 83 | [gi|114386664](http://www.matrixscience.com/cgi/protein_view.pl?file=../data/20091223/FtmmrnHet.dat&hit=gi|114386664&px=1&ave_thresh=53&_sigthreshold=0.05&_server_mudpit_switch=0.001) | Phosphoglycerate kinase (E.C. 2.7.2.3) | 103 | 2 | 42224/5.64 | *Oryza sativa* | All entries | EMP ^20^ |
| 85 | [gi|115458044](http://www.matrixscience.com/cgi/protein_view.pl?file=../data/20091223/FtmmrnHwE.dat&hit=gi|115458044&px=1&ave_thresh=53&_sigthreshold=0.05&_server_mudpit_switch=0.001) | Isocitrate lyase and phosphorylmutase family protein (E.C. 4.1.3.1) | 93 | 3 | 41636/5.66 | *Oryza sativa* | All entries | GAC ^21^ |
| 86 | [gi|2218152](http://www.matrixscience.com/cgi/protein_view.pl?file=../data/20091212/FtmolnatS.dat&hit=gi|2218152&px=1&ave_thresh=52&_sigthreshold=0.05&_server_mudpit_switch=0.001) | Type IIIa membrane protein cp-wap13 | 89 | 2 | 40081/6.24 | *Vigna unguiculata* | All entries | Glycan metabolism ^22^ |
| 88 | [gi|38637314](http://www.matrixscience.com/cgi/protein_view.pl?file=../data/20091212/FtmolnatE.dat&hit=gi|38637314&px=1&ave_thresh=53&_sigthreshold=0.05&_server_mudpit_switch=0.001) | Putative GTP-binding protein | 122 | 2 | 44704/6.30 | *Oryza sativa* | All entries | Signal transduction ^18^ |
| 92 | [gi|2218152](http://www.matrixscience.com/cgi/protein_view.pl?file=../data/20091212/FtmolnatL.dat&hit=gi|2218152&px=1&ave_thresh=52&_sigthreshold=0.05&_server_mudpit_switch=0.001) | Type IIIa membrane protein cp-wap13 | 123 | 2 | 40081/6.24 | *Vigna unguiculata* | All entries | Glycan metabolism ^22^ |
| 94 | [gi|3646373](http://www.matrixscience.com/cgi/protein_view.pl?file=../data/20091203/FtmoracmL.dat&hit=gi|3646373&px=1&ave_thresh=53&_sigthreshold=0.05&_server_mudpit_switch=0.001) | Reversibly glycosylated polypeptide | 193 | 3 | 40079/8.21 | *Oryza sativa* | All entries | Glycan metabolism ^23^ |
| 96 | [gi|115482534](http://www.matrixscience.com/cgi/protein_view.pl?file=../data/20091223/FtmmrGHwm.dat&hit=gi|115482534&px=1&ave_thresh=52&_sigthreshold=0.05&_server_mudpit_switch=0.001) | Cytosolic malate dehydrogenase (E.C. 1.1.1.40) | 172 | 2 | 35888/5.75 | *Oryza sativa* | All entries | Pyruvate metabolism ^24^ |
| 100 | gi|57337458 | Putative malate dehydrogenase (E.C. 1.1.1.37) | 66 | 2 | 30653/9.04 | *Orpinomyces* | Fungi | TCA ^25^ |
| 101 | [gi|115461739](http://www.matrixscience.com/cgi/protein_view.pl?file=../data/20091203/FtmorfEme.dat&hit=gi|115461739&px=1&ave_thresh=53&_sigthreshold=0.05&_server_mudpit_switch=0.001) | 11-S plant seed storage protein family protein | 102 | 2 | 38456/5.81 | *Oryza sativa* | All entries | Storage protein ^26^ |
| 107 | [gi|14192878](http://www.matrixscience.com/cgi/protein_view.pl?file=../data/20091204/FtmooGawm.dat&hit=gi|14192878&px=1&ave_thresh=53&_sigthreshold=0.05&_server_mudpit_switch=0.001) | Stress responsive alpha-beta barrel domain protein | 95 | 2 | 27460/7.07 | *Oryza sativa* | All entries | Stress responsive ^27^ |
| 113 | [gi|54291729](http://www.matrixscience.com/cgi/protein_view.pl?file=../data/20100122/FtmpoxTae.dat&hit=gi|54291729&px=1&ave_thresh=54&_sigthreshold=0.05&_server_mudpit_switch=0.001) | Putative chitinase | 121 | 3 | 32757/6.08 | *Oryza sativa* | All entries | Stress/defense response ^28^ |
| 114 | [gi|115463789](http://www.matrixscience.com/cgi/protein_view.pl?file=../data/20091206/FtmoobuER.dat&hit=gi|115463789&px=1&ave_thresh=53&_sigthreshold=0.05&_server_mudpit_switch=0.001) | Similar to Fructose-bisphosphate aldolase (E.C. 4.1.2.13) | 139 | 2 | 36665/6.56 | *Oryza sativa* | All entries | EMP ^29^ |
| 118 | [gi|115457788](http://www.matrixscience.com/cgi/protein_view.pl?file=../data/20091206/FtmoobuOS.dat&hit=gi|115457788&px=1&ave_thresh=53&_sigthreshold=0.05&_server_mudpit_switch=0.001) | Similar to IN2-2 protein | 128 | 2 | 38495/6.03 | *Oryza sativa* | All entries | Stress/defense response ^30^ |
| 124 | [gi|5777629](http://www.matrixscience.com/cgi/protein_view.pl?file=../data/20091224/FtmmriemL.dat&hit=gi|5777629&px=1&ave_thresh=54&_sigthreshold=0.05&_server_mudpit_switch=0.001) | Peroxidase-like protein (E.C. 1.11.1.-) | 179 | 2 | 37205/6.08 | *Oryza sativa* | All entries | Stress/defense response ^31^ |
| 131 | [gi|1658313](http://www.matrixscience.com/cgi/protein_view.pl?file=../data/20091224/FtmmriESR.dat&hit=gi|1658313&px=1&ave_thresh=53&_sigthreshold=0.05&_server_mudpit_switch=0.001) | Ricin B-related lectin domain containing protein | 168 | 2 | 39146/7.28 | *Oryza sativa* | All entries | Stress responsive ^32^ |
| 132 | gi|974447 | Membrane-bound lytic transglycosylase A precursor | 211 | 3 | 40419/9.04 | *Escherichia coli* | All entries | Glycan metabolism, cell wall recycling ^33^ |
| 138 | [gi|125524074](http://www.matrixscience.com/cgi/protein_view.pl?file=../data/20100125/FtmpmbHaT.dat&hit=gi|125524074&px=1&ave_thresh=52&_sigthreshold=0.05&_server_mudpit_switch=0.001) | Ricin B-related lectin domain containing protein | 90 | 2 | 30949/6.35 | *Oryza sativa* | All entries | Stress responsive ^32^ |
| 144 | gi|125527970 | Quinone reductase 2 | 289 | 3 | 21749/5.86 | *Oryza sativa* | All entries | Stress responsive ^34^ |
| 147 | [gi|115474739](http://www.matrixscience.com/cgi/protein_view.pl?file=../data/20091207/FtmomGsSE.dat&hit=gi|115474739&px=1&ave_thresh=53&_sigthreshold=0.05&_server_mudpit_switch=0.001) | Flavoprotein wrbA | 226 | 3 | 21576/6.08 | *Oryza sativa* | All entries | Stress responsive ^35^ |
| 150 | gi|538430 | Superoxide dismutase (E.C. 1.5.1.1) | 399 | 3 | 15338/5.71 | *Oryza sativa* | All entries | Stress/defense response ^5^ |

Note: ^a)^ The numbering corresponds to the 2-DE gel in figure 4. ^b)^ GI number in NCBI. ^c)^ a unique 4-digit identification number for enzyme identification by the Enzyme Commission (E.C.). ^d)^ MASCOT score of MS/MS. ^e)^ Number of peptides identified by MS/MS. ^f)^ Theoretical molecular weight and *p*I. ^g)^ The used database in the process of MASCOT search. EMP: Embden-Meyerhof pathway. TCA: tricarboxylic acid cycle. GAC: glyoxylic acid cycle. PPP: pentose phosphate pathway.

**Reference**

1. Beinert H, Kennedy MC (1993) Aconitase, a two-faced protein: enzyme and iron regulatory factor. Faseb J 7: 1442-1449.
2. Egli B, Kölling K, Köhler C, Zeeman SC, Streb S (2010) Loss of cytosolic phosphoglucomutase compromises gametophyte development in Arabidopsis. Plant Physiol 154: 1659-1671.
3. Kanamori M, Wixom RL (1963) Studies in valine biosynthesis. V. Characteristics of the purified dihydroxyacid dehydratase from spinach leaves. J Biol Chem 238: 998-1005.
4. Pons G, Raefsky-Estrin C, Carothers DJ, Pepin RA, Javed AA, et al. (1988) Cloning and cDNA sequence of the dihydrolipoamide dehydrogenase component human alpha-ketoacid dehydrogenase complexes. Proc Natl Acad Sci USA 85 : 1422-1426.
5. Alscher RG, Erturk N, Heath LS (2002) Role of superoxide dismutases (SODs) in controlling oxidative stress in plants. J Exp Bot 53: 1331-1341.
6. Cho HY, Kong KH (2005) Molecular cloning, expression, and characterization of a phi-type glutathione S-transferase from *Oryza sativa*. Pestic Biochem Phys 83: 29-36.
7. Udomsinprasert R, Pongjaroenkit S, Wongsantichon J, Oakley AJ, Prapanthadara LA, et al. (2005) Identification, characterization and structure of a new Delta class glutathione transferase isoenzyme. Biochem J 388: 763-771.
8. Whitfield CD, Steers EJ Jr, Weisbach H (1970) Purification and properties of 5-methyltetrahydropteroyltriglutamate-homocysteine transmethylase. J Biol Chem 245: 390-401.
9. Tsutui N, Hirasawa E (2003) Purification and properties of betaine aldehyde dehydrogenase from Avena sativa. J Plant Res 116: 133-140.
10. Chelikani P, Fita I, Loewen PC (2004) Diversity of structures and properties among catalases. Cell Mol Life Sci 61: 192-208.
11. Arfin SM, Umbarger HE (1969) Purification and properties of the acetohydroxy acid isomeroreductase of *Salmonella typhimurium*. J Biol Chem 244: 1118-1127.
12. Maguire M, Coates Anthony RM, Henderson B (2002) Chaperonin 60 unfolds its secrets of cellular communication. Cell Stress Chaperon 7: 317-329.
13. Reed GH, Poyner RR, Larsen TM, Wedekind JE, Rayment I (1996) Structural and mechanistic studies of enolase. Curr Opin Struct Biol 6: 736-743.
14. Birgitta N, Christos T, Beston H, Elzbieta G (1990) On the subunit composition of plant mitochondrial ATP synthase. BBA-Biomembranes 1015: 49-52.
15. Hedstrom L (2009) IMP dehydrogenase: structure, mechanism, and inhibition. Chem Rev 109: 2903-2928.
16. Kaufman AC, Greene CE (1993) Increased alanine transaminase activity associated with tetracycline administration in a cat. J Am Vet Med Assoc 202: 628-630.
17. Bailey-Serres J, Nguyen MT (1992) Purification and characterization of cytosolic 6-phosphogluconate dehydrogenase isozymes from maize. Plant Physiol 100: 1580-1583.
18. Khafizov K, Lattanzi G, Carloni P (2009) G protein inactive and active forms investigated by simulation methods. Proteins: Structure, Function, and Bioinformatics 75: 919-930.
19. Basurko MJ, Marche M, Darriet M, Cassaigne A (1999) Phosphoserine aminotransferase, the second step-catalyzing enzyme for serine biosynthesis. Iubmb Life 48: 525-529.
20. Watson HC, Walker NPC, Shaw PJ, Bryant TN, Wendell PL, et al. (1982) Sequence and structure of yeast phosphoglycerate kinase. Embo J 1: 1635-1640.
21. Beeching JR (1989) High sequence conservation between isocitrate lyase from *Escherichia coli* and *Ricinus communis*. Protein Seq Data Anal 2: 463-466.
22. Chen YM, Chen T, Shen SH, Zheng MZ, Guo YM, et al. (2006) Differential display proteomic analysis of *Picea meyeri* pollen germination and pollen-tube growth after inhibition of actin polymerization by latrunculin B. Plant J 47: 174-195.
23. Dhugga KS, Tiwari SC, Ray PM (1997) A reversibly glycosylated polypeptide (RGP1) possibly involved in plant cell wall synthesis: purification, gene cloning, and trans-Golgi localization. P Natl Acad Sci USA 94: 7679-7684.
24. Leonard AF, José IL, Zafeer ZD, Prakash P, Tatyana B, et al. (1999) Ability of cytosolic malate dehydrogenase and lactate dehydrogenase to increase the ratio of NADPH to NADH oxidation by cytosolic glycerol-3-phosphate dehydrogenase. Arch Biochem Biophys 364: 185-194.
25. Minárik P, Tomásková N, Kollárová M, Antalík M (2002) Malate dehydrogenases-structure and function. Gen Physiol Biophys 21: 257-265.
26. Brinegar C, Goundan S (1993) Isolation and characterization of chenopodin, the 11S seed storage protein of quinoa (*Chenopodium quinoa*). J Agric Food Chem 41: 182-185.
27. Gu R, Fonseca S, Puskas LG, Hackler L Jr, Zvara A, et al. (2004) Transcript identification and profiling during salt stress and recovery of *Populus euphratica*. Tree Physiol 24: 265-276.
28. Salzer P, Bonanomi A, Beyer K, Vögeli-Lange R, Aeschbacher RA, et al. (2000) Differential expression of eight chitinase genes in *Medicago truncatula* roots during mycorrhiza formation, nodulation, and pathogen infection. Mol Plant Microbe Interact 13: 763-777.
29. Konishi H, Yamane H, Maeshima M, Komatsu S (2004) Characterization of fructose-bisphosphate aldolase regulated by gibberellin in roots of rice seedling. Plant Mol Biol 56: 839-848.
30. Hershey HP, Stoner TD (1991) Isolation and characterization of cDNA clones for RNA species induced by substituted benzenesulfonamides in corn. Plant Mol Biol 17: 679-690.
31. Karthikeyan M, V Jayakumar, K Radhika, R Bhaskaran, R Velazhahan, et al. (2005) Induction of resistance in host against the infection of leaf blight pathogen (*Alternaria palandui*) in onion (*Allium cepa* var *aggregatum*). Indian J Biochem Biophys 42: 371-377.

## Shahidi-Noghabi S (2010). Toxicity and mode of action of plant lectins with a ricin-B domain against pest insects. Ghent: Ghent University-Faculty of Bioscience Engineering. 205p.

1. Suvorov M, Lee M, Hesek D, Boggess B, Mobashery S (2008) Lytic transglycosylase MltB of Escherichia coli and its role in recycling of peptidoglycan strands of bacterial cell wall. J Am Chem Soc130: 11878-11879.
2. Hsieh TC, Wang Z, Hamby CV, Wu JM (2005) Inhibition of melanoma cell proliferation by resveratrol is correlated with upregulation of quinone reductase 2 and p53. Biochem Biophys Res Comm 334: 223-230.
3. Wolfova J, Grandori R, Kozma E, Chatterjee N, Carey J, et al. (2005) Crystallization of the flavoprotein WrbA optimized by using additives and gels. J Cryst Growth 284: 502-505.
