# Supplementary material for: Comparative Metaproteomic Analysis on Consecutively Rehmannia glutinosa-Monocultured Rhizosphere Soil
Source: PLoS One. 2011 May 31;6(5):e20611. doi: 10.1371/journal.pone.0020611 (PMC3105091; doi:10.1371/journal.pone.0020611)
Supplement: Table S3 — Proteins identified by MS. (DOC) [file pone.0020611.s004.doc]

**Table S3.** Proteins identified by MS.

| Spot no. ^a)^ | GI no.^b)^ | Protein name  (Identification number ^c)^) | Score ^d)^ | PMF  /converage ^e)^ | MW/ p*I*^f)^ | Species | Database^g)^ | Function |
| --- | --- | --- | --- | --- | --- | --- | --- | --- |
| 19 | gi|115447403 | Phenylalanine ammonia-lyase (E.C. 4.3.1.24) | 135 | 14/26% | 76021/6.07 | *Oryza sativa* | All entries | Secondary metabolism ^1^ |
| 20 | gi|254818358 | COG0142 Geranylgeranyl pyrophosphate synthase (E.C. 2.5.1.29) | 81 | 10/33% | 36146/4.87 | *Mycobacterium intracellulare* | Bacteria | Secondary metabolism ^2^ |
| 21 | gi|115467154 | Annexin p33 | 322 | 6/18% | 35984/6.21 | *Oryza sativa* | All entries | Signal transduction ^3^ |
| 45 | gi|254444928 | Threonyl-tRNA synthetase (E.C. 6.1.1.3) | 80 | 10/29% | 70057/5.15 | *Verrucomicrobiae bacterium* | Bacteria | Protein metabolism ^4^ |
| 50 | gi|257466736 | Filamentous hemagglutinin outer membrane protein | 85 | 14/47% | 40650/9.16 | *Fusobacterium gonidiaformans* | Bacteria | Virulence factor ^5^ |
| 53 | gi|148977392 | Cellulose synthase regulator protein | 83 | 11/25% | 78615/4.57 | *Vibrionales bacterium* | Bacteria | Glycan metabolism ^6^ |
| 62 | gi|94987559 | Chemotaxis signal transduction protein | 85 | 7/32% | 35853/4.93 | *Lawsonia intracellularis* | Bacteria | Signal transduction ^7^ |
| 68 | gi|255727695 | Mitochondrial ribosomal protein L8 | 82 | 9/45% | 29860/9.25 | *Candida tropicalis* | Fungi | Mitochondrial protein metabolism ^8^ |
| 69 | gi|117625497 | P-loop ATPase | 86 | 12/48% | 32554/6.73 | *Escherichia coli* | Bacteria | Energy metabolism ^9^ |
| 70 | gi|92112843 | Phosphate transport regulator | 88 | 8/61% | 25660/4.94 | *Chromohalobacter salexigens* | Bacteria | Signal transduction ^10^ |
| 72 | gi|260945048 | G-protein signaling regulator | 73 | 7/29% | 49207/7.25 | *Clavispora lusitaniae* | Fungi | Signal transduction ^11^ |
| 73 | gi|192290036 | ABC transporter, ATP-binding protein | 88 | 8/49% | 34941/5.76 | *Rhodopseudomonas palustris* | Bacteria | Membrane transport ^12^ |
| 91 | gi|100801668 | S-adenosylmethionine synthetase (E.C. 2.5.1.6) | 163 | 17/57% | 42979/5.74 | *Oryza rufipogon* | All entries | Amino acid metabolism ^13^ |
| 104 | gi|255727695 | Mitochondrial ribosomal protein L8 | 80 | 11/56% | 29860/9.25 | *Candida tropicalis* | Fungi | Mitochondrial protein metabolism ^8^ |
| 108 | gi|255727695 | Mitochondrial ribosomal protein L8 | 89 | 14/63% | 29860/9.25 | *Candida tropicalis* | Fungi | Mitochondrial protein metabolism ^8^ |
| 111 | gi|121601839 | Lipoyl synthase (E.C. 2.8.1.8) | 87 | 13/36% | 35937/8.80 | *Bartonella bacilliformis* | Bacteria | Metabolism of Cofactors and Vitamins ^14^ |
| 115 | gi|255727695 | Mitochondrial ribosomal protein L8 | 86 | 10/52% | 29860/9.25 | *Candida tropicalis* | Fungi | Mitochondrial protein metabolism ^8^ |
| 119 | gi|170103931 | Glycosyltransferase family 39 protein | 74 | 11/33% | 43350/8.36 | *Laccaria bicolor* | Fungi | Protein metabolism ^15^ |
| 120 | gi|255727695 | Mitochondrial ribosomal protein L8 | 77 | 10/52% | 29860/9.25 | *Candida tropicalis* | Fungi | Mitochondrial Protein metabolism ^8^ |
| 123 | gi|291184583 | Actin patches distal protein 1 | 74 | 11/36% | 33458/6.79 | *Trichophyton verrucosum* | Fungi | Stress/defense response ^16^ |
| 126 | gi|115452789 | Ricin B-related lectin domain containing protein | 150 | 18/54% | 39254/6.30 | *Oryza sativa* | All entries | Stress/defense response ^17^ |
| 128 | gi|50554495 | Ubiquitin carrier protein | 74 | 10/28% | 38356/9.71 | *Yarrowia lipolytica* | Fungi | Protein metabolism ^18^ |
| 133 | gi|145234049 | Peptidase S15 | 72 | 7/45% | 17840/10.25 | *Aspergillus niger* | Fungi | Protein metabolism ^19^ |
| 134 | gi|75762505 | Tellurite resistance protein | 85 | 10/44% | 27371/6.54 | *Bacillus thuringiensis serovar israelensis* | Bacteria | Xenobiotics Metabolism ^20^ |
| 135 | gi|255727695 | Mitochondrial ribosomal protein L8 | 83 | 12/51% | 29860/9.25 | *Candida tropicalis* | Fungi | Mitochondrial protein metabolism ^8^ |
| 137 | gi|242804562 | Pentalenene synthase (E.C. 4.2.3.7) | 76 | 7/30% | 21332/4.90 | *Talaromyces stipitatus* | Fungi | Secondary metabolism ^21^ |
| 145 | gi|118588752 | Methyl-accepting chemotaxis protein | 95 | 28/19% | 207084/4.66 | *Stappia aggregata* | Bacteria | Signal transduction ^22^ |
| 151 | gi|115484359 | Pollen-specific desiccation-associated LLA23 protein | 106 | 8/66% | 15456/6.20 | *Oryza sativa* | All entries | Stress/defense response ^23^ |

Note: ^a)^ The numbering corresponds to the 2-DE gel in figure 4. ^b)^ GI number in NCBI. ^c)^ a unique 4-digit identification number for enzyme identification by the Enzyme Commission (E.C.). ^d)^ MASCOT score of MS. ^e)^ The number of peptides identified by MS/sequence percentage coverage. ^f)^ Theoretical molecular weight and *p*I. ^g)^ The used database in the process of MASCOT search. EMP: Embden-Meyerhof pathway. PPP: pentose phosphate pathway.

**Reference**

1. Elkind [Y](http://www.pnas.org/search?author1=Y+Elkind&sortspec=date&submit=Submit), Edwards [R](http://www.pnas.org/search?author1=R+Edwards&sortspec=date&submit=Submit), Mavandad [M](http://www.pnas.org/search?author1=M+Mavandad&sortspec=date&submit=Submit), Hedrick [SA](http://www.pnas.org/search?author1=S+A+Hedrick&sortspec=date&submit=Submit), Ribak [O](http://www.pnas.org/search?author1=O+Ribak&sortspec=date&submit=Submit), et al. (1990) Abnormal plant development and down-regulation of phenylpropanoid biosynthesis in transgenic tobacco containing a heterologous phenylalanine ammonia-lyase gene. P Natl Acad Sci USA 87: 9057-9061.
2. Sagami H, Ogura K (1981) Geranylgeranyl pyrophosphate synthetase lacking geranyl-transferring activity from *Micrococcus luteus*. J Biochem 89: 1573-1580.
3. Breton G, Vazquez-Tello A, Danyluk J, Sarhan F (2000) Two novel intrinsic annexins accumulate in wheat membranes in response to low temperature. Plant Cell Physiol 41: 177-184.
4. **Rajan S, Anne-Catherine DB,** **Pascale R,** **Joel C,** **Mathias S, et al. (**1999) The structure of threonyl-tRNA synthetase-tRNA^Thr^ complex enlightens its repressor activity and reveals an essential zinc ion in the active site. Cell 97: 371-381.
5. Gottig N, Garavaglia BS, Garofalo CG, Orellano EG, Ottado J (2009) A filamentous hemagglutinin-like protein of *Xanthomonas axonopodis* pv. citri, the phytopathogen responsible for citrus canker, is involved in bacterial virulence. PLoS One 4: e4358.
6. Blum M, Boehler M, Randall E, Young V, Csukai M, et al. (2010) Mandipropamid targets the cellulose synthase-like PiCesA3 to inhibit cell wall biosynthesis in the oomycete plant pathogen, Phytophthora infestans. Mol Plant Pathol 11: 227-243.
7. Baker MD, Wolanin PM, Stock JB (2005) Signal transduction in bacterial chemotaxis. Bioessays 28: 9-22.
8. Kitakawa M, Grohmann L, Graack HR, Isono K (1990) Cloning and characterization of nuclear genes for two mitochondrial ribosomal proteins in *Saccharomyces cerevisiae*. Nucleic Acids Res 18: 1521-1529.
9. Iyer LM, Makarova KS, Koonin EV, Aravind L (2004) Comparative genomics of the FtsK-HerA superfamily of pumping ATPases: implications for the origins of chromosome segregation, cell division and viral capsid packaging. Nucleic Acids Res 32: 5260-5279.
10. Oganesyan V, Oganesyan N, Adams PD, Jancarik J, Yokota HA, et al. (2005) Crystal structure of the "PhoU-like" phosphate uptake regulator from *Aquifex aeolicus*. J Bacteriol 187: 4238-4244.
11. De Vries L, Farquhar MG, Zheng B, Fischer T, Elenko E (2000) The regulator of G protein signaling family. Annu Rev Pharmacol Toxicol 40: 235–271.
12. Davidson AL, Chen J. (2004). ATP-binding cassette transporters in bacteria. Annu Rev Biochem 73: 241–268.
13. Frank VB, Rudy D, Jan G, Marc VM, Allan C (1994) Characterization of a s-adenosylmethionine synthetase gene in rice. Plant Physiol 105: 1463-1464.
14. Cicchillo RM, Iwig DF, Jones AD, Nesbitt NM, Baleanu-Gogonea C, et al. (2004) Lipoyl synthase requires two equivalents of S-adenosyl-L-methionine to synthesize one equivalent of lipoic acid. Biochemistry 43: 6378-6386.
15. Lommel M, Strahl S (2009) Protein O-mannosylation: conserved from bacteria to humans. Glycobiology 19: 816-828.
16. Entian KD, Schuster T, Hegemann JH, Becher D, Feldmann H, et al. (1999) Functional analysis of 150 deletion mutants in *Saccharomyces cerevisiae* by a systematic approach. Mol Gen Genet. 262: 683-702.

## Shahidi-Noghabi S (2010). Toxicity and mode of action of plant lectins with a ricin-B domain against pest insects. Ghent: Ghent University-Faculty of Bioscience Engineering. 205p.

1. Aristarkhov A, Eytan E, Moghe A, Admon A, Hershko A, et al. (1996) E2-C, a cyclin-selective ubiquitin carrier protein required for the destruction of mitotic cyclins. Proc Natl Acad Sci USA 93: 4294-4299.
2. Rawlings ND, Barrett AJ (1993) Evolutionary families of peptidases. Biochem J 290: 205-218.
3. O’Gara JP, Gomelsky M, Kaplan S (1997) Identification and molecular genetic analysis of multiple loci contributing to high-level tellurite resistance in *Rhodobacter sphaeroides* 2.4.1. Appl Environ Microbiol 63: 4713-4720.
4. Seemann M, Zhai G, de Kraker JW, Paschall CM, Christianson DW, et al. (2002) Pentalenene synthase. Analysis of active site residues by site-directed mutagenesis. J Am Chem Soc 124: 7681-7689.
5. Yost CK, Clark KT, Del Bel KL, Hynes MF (2003) Characterization of the nodulation plasmid encoded chemoreceptor gene mcpG from *Rhizobium leguminosarum*. BMC Microbiol 3: 1.
6. Huang JC, Lin SM, Wang CS (2000) A pollen-specific and desiccation-associated transcript in *Lilium longiflorum* during development and stress. Plant Cell Physiol 41: 477-485.
