# Supplementary material for: Comparative Metaproteomic Analysis on Consecutively Rehmannia glutinosa-Monocultured Rhizosphere Soil
Source: PLoS One. 2011 May 31;6(5):e20611. doi: 10.1371/journal.pone.0020611 (PMC3105091; doi:10.1371/journal.pone.0020611)
Supplement: Table S4 — Differentially expressed proteins identified by MS/MS. (DOC) [file pone.0020611.s005.doc]

**Table S4. Differentially expressed proteins identified by MS/MS.**

| Spot no. ^a)^ | GI no.^b)^ | Protein name  (Identification number ^c)^) | Score  (PMF) ^d)^ | PMF/  converage ^e)^ | MW/ p*I*^f)^ | Score  (MS-MS) ^g)^ | Pept ^h)^ | Species | Database ^i)^ | Function |
| --- | --- | --- | --- | --- | --- | --- | --- | --- | --- | --- |
| 1 | [gi|75225211](http://www.matrixscience.com/cgi/protein_view.pl?file=../data/20100121/Ftmponuwh.dat&hit=1) | Putative aconitate hydratase (E.C. 4.2.1.3) | 107 | 23/30% | 98591/5.67 | 189 | 4 | *Oryza sativa* | All entries | TCA, GAC ^1^ |
| [2](http://www.matrixscience.com/cgi/protein_view.pl?file=../data/20100121/FtmponsOh.dat&hit=2) | gi|115450595 | Aconitate hydratase (E.C. 4.2.1.3) | 146 | 25/32% | 106862/6.45 | 239 | 4 | *Oryza sativa* | All entries | TCA, GAC ^1^ |
| 3 | [gi|162461914](http://www.matrixscience.com/cgi/protein_view.pl?file=../data/20100118/FtmpffaOE.dat&hit=gi|162461914&px=1&ave_thresh=53&_sigthreshold=0.05&_server_mudpit_switch=0.001) | Phenylalanine ammonia-lyase (E.C. 4.3.1.24) | 78 | 15/24% | 75336/6.52 | 269 | 3 | *Zea mays* | All entries | Secondary metabolism ^2^ |
| 4 | gi|108862992 | 5-methyltetrahydropteroyltriglutamate-homocysteine methyltransferase (E.C. 2.1.1.14) | 120 | 16/25% | 84925/5.93 | 437 | 5 | *Oryza sativa* | All entries | Amino acid metabolism ^3^ |
| 6 | [gi|110288669](http://www.matrixscience.com/cgi/protein_view.pl?file=../data/20100118/Ftmpffctt.dat&hit=3) | Putative enolase (E.C. 4.2.1.11) | 215 | 23/61% | 51834/5.84 | 353 | 2 | *Oryza sativa* | All entries | EMP ^4^ |
| 7 | [gi|115470967](http://www.matrixscience.com/cgi/protein_view.pl?file=../data/20100118/FtmpffcOt.dat&hit=2) | Methylmalonate-semialdehyde dehydrogenase (E.C. 1.2.1.127) | 92 | 23/53% | 57666/5.98 | 393 | 4 | *Oryza sativa* | All entries | Amino acid metabolism ^5^ |
| 8 | gi|115467370 | Pyrophosphate-dependent 6-phosphofructose-1-kinase (E.C. 2.7.1.11) | 95 | 15/34% | 61907/6.01 | 166 | 3 | *Oryza sativa* | All entries | EMP ^6^ |
| 9 | [gi|115459078](http://www.matrixscience.com/cgi/protein_view.pl?file=../data/20100106/FtmmSxene.dat&hit=gi|115459078&px=1&ave_thresh=53&_sigthreshold=0.05&_server_mudpit_switch=0.001) | Glyceraldehyde-3-phosphate dehydrogenase (E.C. 1.2.1.9) | 164 | 20/64% | 36921/6.34 | 369 | 3 | *Oryza sativa* | All entries | EMP ^7^ |
| 10 | [gi|3024122](http://www.matrixscience.com/cgi/protein_view.pl?file=../data/20100121/FtmponTmE.dat&hit=gi|3024122&px=1&ave_thresh=53&_sigthreshold=0.05&_server_mudpit_switch=0.001) | S-adenosylmethionine synthetase (E.C. 2.5.1.6) | 131 | 17/52% | 43330/5.68 | 392 | 4 | *Oryza sativa* | All entries | Amino acid metabolism ^8^ |
| 11 | [gi|51536102](http://www.matrixscience.com/cgi/protein_view.pl?file=../data/20091206/FtmooecaO.dat&hit=1) | Putative formate-tetrahydrofolate ligase (E.C. 6.3.4.3) | 86 | 15/25% | 68639/6.55 | 122 | 3 | *Oryza sativa* | All entries | One carbon pool ^9^ |
| 12 | [gi|115455349](http://www.matrixscience.com/cgi/protein_view.pl?file=../data/20100118/FtmpffuOO.dat&hit=gi|115455349&px=1&ave_thresh=53&_sigthreshold=0.05&_server_mudpit_switch=0.001) | Exoglucanase precursor (E.C. 3.2.1.91) | 125 | 19/36% | 68191/7.23 | 189 | 3 | *Oryza sativa* | All entries | Glycan metabolism ^10^ |
| 13 | [gi|115485405](http://www.matrixscience.com/cgi/protein_view.pl?file=../data/20100121/FtmponsTO.dat&hit=2) | Serine hydroxymethyltransferase (E.C. 2.1.2.1) | 78 | 18/36% | 51799/7.16 | 130 | 2 | *Oryza sativa* | All entries | Amino acid metabolism ^11^ |
| 14 | gi|115450567 | Glutathione S-transferase GSTF15 (E.C. 2.5.1.18) | 89 | 7/23% | 25755/6.67 | 255 | 4 | *Oryza sativa* | All entries | Xenobiotics Metabolism ^12^, Amino acid metabolism ^13^ |
| 23 | gi|54606800 | NADP dependent malic enzyme (E.C. 1.1.1.40) | 172 | 25/38% | 65824/5.79 | 346 | 4 | *Oryza sativa* | All entries | Pyruvate metabolism ^14^ |
| 46 | gi|115470967 | similar to Methylmalonate-semialdehyde dehydrogenase (E.C. 1.2.1.127) | 109 | 22/46% | 57666/5.98 | 379 | 4 | *Oryza sativa* | All entries | Amino acid metabolism ^5^ |
| 5 | [gi|115454931](http://www.matrixscience.com/cgi/protein_view.pl?file=../data/20100118/FtmpffTmS.dat&hit=gi|115454931&px=1&ave_thresh=52&_sigthreshold=0.05&_server_mudpit_switch=0.001) | Phosphoglucomutase (E.C. 5.4.2.2.) |  |  | 63138/5.4 | 85 | 2 | *Oryza sativa* | All entries | EMP, PPP ^15^ |
| 15 | [gi|115477815](http://www.matrixscience.com/cgi/protein_view.pl?file=../data/20100121/FtmporTnO.dat&hit=gi|115477815&px=1&ave_thresh=53&_sigthreshold=0.05&_server_mudpit_switch=0.001) | Similar to Dihydroxy-acid dehydratase (E.C. 4.2.1.9) |  |  | 64237/7.12 | 298 | 4 | *Oryza sativa* | All entries | Amino acid metabolism ^16^ |
| 16 | [gi|115436320](http://www.matrixscience.com/cgi/protein_view.pl?file=../data/20091207/FtmomGaaO.dat&hit=gi|115436320&px=1&ave_thresh=52&_sigthreshold=0.05&_server_mudpit_switch=0.001) | Dihydrolipoamide dehydrogenase family protein (E.C. 1.8.1.4) |  |  | 53009/7.21 | 69 | 2 | *Oryza sativa* | All entries | TCA ^17^ |
| 17 | [gi|115436024](http://www.matrixscience.com/cgi/protein_view.pl?file=../data/20100118/FtmpffsSt.dat&hit=gi|115436024&px=1&ave_thresh=54&_sigthreshold=0.05&_server_mudpit_switch=0.001) | Superoxide dismutase [Mn] (E.C. 1.15.1.1) |  |  | 22634/7.77 | 331 | 4 | *Oryza sativa* | All entries | Stress/defense response ^18^ |
| 18 | [gi|125542289](http://www.matrixscience.com/cgi/protein_view.pl?file=../data/20100118/FtmpffunR.dat&hit=gi|125542289&px=1&ave_thresh=53&_sigthreshold=0.05&_server_mudpit_switch=0.001) | Glutathione S-transferase (E.C. 2.5.1.18) |  |  | 25575/6.72 | 208 | 2 | *Oryza sativa* | All entries | Xenobiotics Metabolism ^12^, Amino acid metabolism ^13^ |
| 33 | gi|3868754 | Catalase (E.C. 1.11.1.6) |  |  | 57052/6.49 | 147 | 2 | *Oryza sativa* | All entries | Stress/defense response ^19^ |
| 39 | gi|115482382 | mitochondrial chaperonin-60 |  |  | 61097/5.71 | 204 | 4 | *Oryza sativa* | All entries | Protein folding ^20^ |
| 60 | [gi|3925239](http://www.matrixscience.com/cgi/protein_view.pl?file=../data/20100126/FtmpSiawT.dat&hit=gi|3925239&px=1&ave_thresh=53&_sigthreshold=0.05&_server_mudpit_switch=0.001) | 6-phosphogluconate dehydrogenase isoenzyme A (E.C. 1.1.1.44) |  |  | 19147/5.24 | 85 | 2 | *Zea mays* | All entries | PPP ^21^ |
| 131 | [gi|1658313](http://www.matrixscience.com/cgi/protein_view.pl?file=../data/20091224/FtmmriESR.dat&hit=gi|1658313&px=1&ave_thresh=53&_sigthreshold=0.05&_server_mudpit_switch=0.001) | Ricin B-related lectin domain containing protein |  |  | 39146/7.28 | 168 | 2 | *Oryza sativa* | All entries | Stress responsive^22^ |
| 19 | gi|115447403 | Phenylalanine ammonia-lyase (E.C. 4.3.1.24) | 135 | 14/26% | 76021/6.07 |  |  | *Oryza sativa* | All entries | Secondary metabolism ^2^ |
| 20 | gi|254818358 | COG0142 Geranylgeranyl pyrophosphate synthase (E.C. 2.5.1.29) | 81 | 10/33% | 36146/4.87 |  |  | *Mycobacterium intracellulare* | Bacteria | Secondary metabolism ^23^ |
| 21 | gi|115467154 | Annexin p33 | 322 | 6/18% | 35984/6.21 |  |  | *Oryza sativa* | All entries | Signal transduction ^24^ |
| 45 | gi|254444928 | threonyl-tRNA synthetase (E.C. 6.1.1.3) | 80 | 10/29% | 70057/5.15 |  |  | *Verrucomicrobiae bacterium* | Bacteria | Protein metabolism ^25^ |
| 50 | gi|257466736 | Filamentous hemagglutinin outer membrane protein | 85 | 14/47% | 40650/9.16 |  |  | *Fusobacterium gonidiaformans* | Bacteria | Virulence factor ^26^ |
| 53 | gi|148977392 | cellulose synthase regulator protein | 83 | 11/25% | 78615/4.57 |  |  | *Vibrionales bacterium* | Bacteria | Cell wall biosynthesis ^27^ |
| 111 | gi|121601839 | lipoyl synthase (E.C. 2.8.1.8) | 87 | 13/36% | 35937/8.80 |  |  | *Bartonella bacilliformis* | Bacteria | Metabolism of Cofactors and Vitamins ^28^ |
| 135 | gi|255727695 | Mitochondrial ribosomal protein L8 | 83 | 12/51% | 29860/9.25 |  |  | *Candida tropicalis* | Fungi | Mitochondrial protein metabolism ^29^ |
| 145 | gi|118588752 | Methyl-accepting chemotaxis protein | 95 | 28/19% | 207084/4.66 |  |  | *Stappia aggregata* | Bacteria | Signal transduction ^30^ |

Note: Protein spots 1-14, 23 and 46 shared equal searching by MS/MS and MS. Protein spots 15-18, 33, 39, 60 and 131 matched at least two MS/MS peptides. The remainders matched at least three PMFs. ^a)^ The numbering corresponds to the 2-DE gel in figure 4. ^b)^ GI number in NCBI. ^c)^ a unique 4-digit identification number for enzyme identification by the Enzyme Commission (E.C.). ^d)^ MASCOT score of PMF. ^e)^ The number of peptides identified by MS/sequence percentage coverage. ^f)^ Theoretical molecular weight and p*I*. ^g)^ MASCOT score of MS/MS. ^h)^ Number of peptides identified by MS/MS. ^i)^ The used database in the process of MASCOT search. EMP: Embden-Meyerhof pathway. TCA: tricarboxylic acid cycle. GAC: glyoxylic acid cycle. PPP: pentose phosphate pathway.

**Reference**

1. Beinert H, Kennedy MC (1993) Aconitase, a two-faced protein: enzyme and iron regulatory factor. Faseb J 7: 1442-1449.
2. Elkind [Y](http://www.pnas.org/search?author1=Y+Elkind&sortspec=date&submit=Submit), Edwards [R](http://www.pnas.org/search?author1=R+Edwards&sortspec=date&submit=Submit), Mavandad [M](http://www.pnas.org/search?author1=M+Mavandad&sortspec=date&submit=Submit), Hedrick [SA](http://www.pnas.org/search?author1=S+A+Hedrick&sortspec=date&submit=Submit), Ribak [O](http://www.pnas.org/search?author1=O+Ribak&sortspec=date&submit=Submit), et al. (1990) Abnormal plant development and down-regulation of phenylpropanoid biosynthesis in transgenic tobacco containing a heterologous phenylalanine ammonia-lyase gene. P Natl Acad Sci USA 87: 9057-9061.
3. Whitfield CD, Steers EJ Jr, Weisbach H (1970) Purification and properties of 5-methyltetrahydropteroyltriglutamate-homocysteine transmethylase. J Biol Chem 245: 390-401.
4. Reed GH, Poyner RR, Larsen TM, Wedekind JE, Rayment I (1996) Structural and mechanistic studies of enolase. Curr Opin Struct Biol 6: 736-743.
5. Naoki T, Hideyuki T, Hidemi K, Makoto M, Shoichiro A, et al. (2005) Proteome approach to characterize the methylmalonate-semialdehyde dehydrogenase that is regulated by gibberellin. J Proteome Res 4: 1575-1582.
6. Suzuki J, Mutton MA, Ferro MI, Lemos MV, Pizauro JM, et al. (2003) Putative pyrophosphate phosphofructose 1-kinase genes identified in sugar cane may be getting energy from pyrophosphate. Genet Mol Res 2: 376-382.
7. Michael AS (1999) New insights into an old protein: the functional diversity of mammalian glyceraldehyde-3-phosphate dehydrogenase. BBA-Biomembranes 1432: 159-184.
8. Frank VB, Rudy D, Jan G, Marc VM, Allan C (1994) Characterization of a s-adenosylmethionine synthetase gene in rice. Plant Physiol 105: 1463-1464.
9. Marx CJ, Laukel M, Vorholt JA, Lidstrom ME (2003) Purification of the formate-tetrahydrofolate ligase from methylobacterium extorquens AM1 and demonstration of its requirement for methylotrophic growth. J Bacteriol 185: 7169-7175.
10. Han Y, Chen H (2010) Biochemical characterization of a maize stover beta-exoglucanase and its use in lignocellulose conversion. Bioresour Technol 101: 6111-6117.
11. Ogawa H, Gomi T, Fujioka M (2000) Serine hydroxymethyltransferase and threonine aldolase: are they identical? Int J Biochem Cell Biol 32: 289-301.
12. Cho HY, Kong KH (2005) Molecular cloning, expression, and characterization of a phi-type glutathione S-transferase from *Oryza sativa*. Pestic Biochem Phys 83: 29-36.
13. Udomsinprasert R, Pongjaroenkit S, Wongsantichon J, Oakley AJ, Prapanthadara LA, et al. (2005) Identification, characterization and structure of a new Delta class glutathione transferase isoenzyme. Biochem J 388: 763-771.
14. Rothermel BA, Nelson T (1989) Primary structure of the maize NADP-dependent malic enzyme. J Biol Chem, 264: 19587-19592.
15. Egli B, Kölling K, Köhler C, Zeeman SC, Streb S (2010) Loss of cytosolic phosphoglucomutase compromises gametophyte development in Arabidopsis. Plant Physiol 154: 1659-1671.
16. Kanamori M, Wixom RL (1963) Studies in valine biosynthesis. V. Characteristics of the purified dihydroxyacid dehydratase from spinach leaves. J Biol Chem 238: 998-1005.
17. Pons G, Raefsky-Estrin C, Carothers DJ, Pepin RA, Javed AA, et al. (1988) Cloning and cDNA sequence of the dihydrolipoamide dehydrogenase component human alpha-ketoacid dehydrogenase complexes. Proc Natl Acad Sci USA 85 : 1422-1426.
18. Alscher RG, Erturk N, Heath LS (2002) Role of superoxide dismutases (SODs) in controlling oxidative stress in plants. J Exp Bot 53: 1331-1341.
19. Chelikani P, Fita I, Loewen PC (2004) Diversity of structures and properties among catalases. Cell Mol Life Sci 61: 192-208.
20. Maguire M, Coates Anthony RM, Henderson B (2002) Chaperonin 60 unfolds its secrets of cellular communication. Cell Stress Chaperon 7: 317-329.
21. Bailey-Serres J, Nguyen MT (1992) Purification and characterization of cytosolic 6-phosphogluconate dehydrogenase isozymes from maize. Plant Physiol 100: 1580-1583.

## Shahidi-Noghabi S (2010). Toxicity and mode of action of plant lectins with a ricin-B domain against pest insects. Ghent: Ghent University-Faculty of Bioscience Engineering. 205p.

1. Sagami H, Ogura K (1981) Geranylgeranyl pyrophosphate synthetase lacking geranyl-transferring activity from *Micrococcus luteus*. J Biochem 89: 1573-1580.
2. Breton G, Vazquez-Tello A, Danyluk J, Sarhan F (2000) Two novel intrinsic annexins accumulate in wheat membranes in response to low temperature. Plant Cell Physiol 41: 177-184.
3. **Rajan S, Anne-Catherine DB,** **Pascale R,** **Joel C,** **Mathias S, et al. (**1999) The structure of threonyl-tRNA synthetase-tRNA^Thr^ complex enlightens its repressor activity and reveals an essential zinc ion in the active site. Cell 97: 371-381.
4. Gottig N, Garavaglia BS, Garofalo CG, Orellano EG, Ottado J (2009) A filamentous hemagglutinin-like protein of *Xanthomonas axonopodis* pv. citri, the phytopathogen responsible for citrus canker, is involved in bacterial virulence. PLoS One 4: e4358.
5. Blum M, Boehler M, Randall E, Young V, Csukai M, et al. (2010) Mandipropamid targets the cellulose synthase-like PiCesA3 to inhibit cell wall biosynthesis in the oomycete plant pathogen, Phytophthora infestans. Mol Plant Pathol 11: 227-243.
6. Cicchillo RM, Iwig DF, Jones AD, Nesbitt NM, Baleanu-Gogonea C, et al. (2004) Lipoyl synthase requires two equivalents of S-adenosyl-L-methionine to synthesize one equivalent of lipoic acid. Biochemistry 43: 6378-6386.
7. Kitakawa M, Grohmann L, Graack HR, Isono K (1990) Cloning and characterization of nuclear genes for two mitochondrial ribosomal proteins in *Saccharomyces cerevisiae*. Nucleic Acids Res 18: 1521-1529.
8. Yost CK, Clark KT, Del Bel KL, Hynes MF (2003) Characterization of the nodulation plasmid encoded chemoreceptor gene mcpG from *Rhizobium leguminosarum*. BMC Microbiol 3: 1.
